# Extracellular vesicle miRNAs promote the intestinal microenvironment by interacting with microbes in colitis

**DOI:** 10.1080/19490976.2022.2128604

**Published:** 2022-09-29

**Authors:** Qichen Shen, Zhuizui Huang, Lingyan Ma, Jiachen Yao, Ting Luo, Yao Zhao, Yingping Xiao, Yuanxiang Jin

**Affiliations:** a Department of Biotechnology, College of Biotechnology and Bioengineering, Zhejiang University of Technology, Hangzhou, China; b State Key Laboratory for Managing Biotic and Chemical Threats to the Quality and Safety of Agro-Products, Institute of Agro-Product Safety and Nutrition, Zhejiang Academy of Agricultural Sciences, Hangzhou, China; c Health Informatics Centre, Department of Learning, Informatics, Management and Ethics, Karolinska Institute, Stockholm, Sweden

**Keywords:** Extracellular vesicles, membrane vesicles, miRNA, colitis, microbiota

## Abstract

Inflammatory bowel disease (IBD) is a global disease with no cure. Disruption of the microbial ecosystem is considered to be an important cause of IBD. Extracellular vesicles (EVs) are vital participants in cell–cell and cell-organism communication. Both host-derived EVs and bacteria-derived membrane vesicles (OMVs) contribute to homeostasis in the intestine. However, the roles of EVs-miRNAs and MVs in host-microbe interactions in colitis remain unclear. In the present study, the animal model of colitis was established by dextran sulfate sodium (DSS) to investigate the changes of miRNAs in colonic EVs from colitis. Several miRNAs were significantly altered in colitis EVs. miR-181b-5p transplantation inhibited M1 macrophage polarization and promoted M2 polarization to reduce the levels of inflammation both in acute and remission of chronic colitis. miR-200b-3p could interact with bacteria and regulate the composition of the microbiota, which contributed to intestinal barrier integrity and homeostasis. Notably, MVs from normal feces could effectively reverse the composition of the intestinal microbiota, restore the intestinal barrier and rescue colitis, and BMVs from colitis would also have similar effects after miR-200b-3p treatment. Our results preliminarily identify a vesicle-based host‐microbe interaction cycle in colitis and provide new ideas for colitis treatment.

## Graphical Abstract

**Figure:**
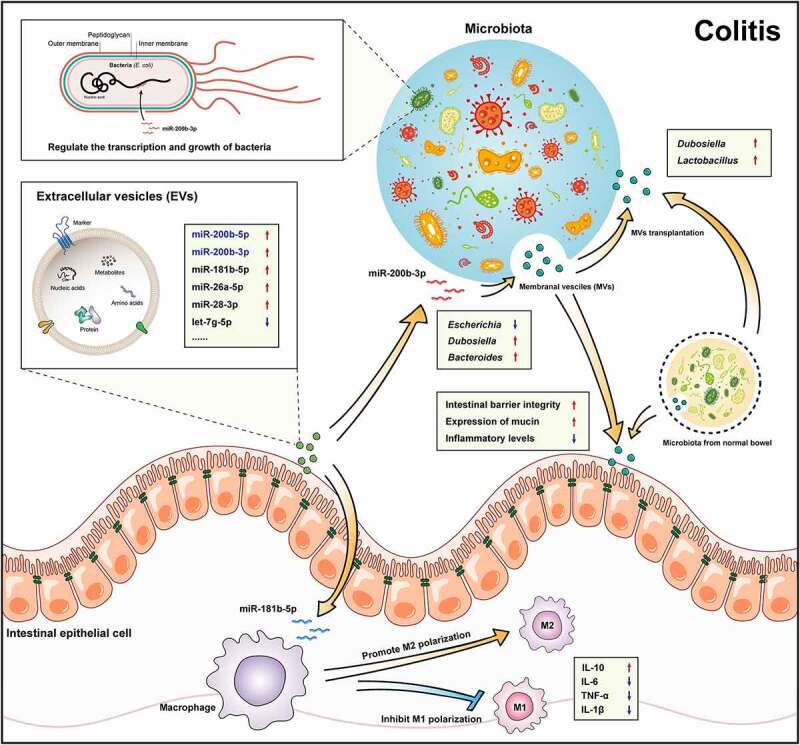

## 1 Introduction

At present, inflammatory bowel disease (IBD) is increasing in incidence worldwide and affects more than 10 million people according to the European Federation of Crohn´s and Ulcerative Colitis Associations. IBD is a chronic inflammatory condition of the gastrointestinal (GI) tract with complex etiology and pathogenesis that can be divided into two main subtypes: ulcerative colitis (UC) and Crohn’s disease (CD).^1^ UC is a lifelong disease with no cure. Several such investigations have shown an increased risk of UC in Asia and Latin America.^2,3^ Although the triggers for UC continue to elude researchers, this inflammatory condition is thought to result from disruption of the microbial ecosystem in the colon,^4,5^ including disruption of the colonic microenvironment,^6^ epithelial barrier and mucosa.^7^ Currently, the advanced treatment approaches for UC are related to inhibiting the immune cell migration,^8^ promoting epithelial barrier repair and fecal microbiota transplantation (FMT).^9,10^ Extracellular vesicles (EVs) were initially described nearly 35 years ago and they play a crucial role in cell-to-cell communication.^11^ Exosomes (small EVs, sEVs), which are a subtype of EVs, are ~40 to ~200 nm (or ~30 to ~150 nm), single-membranes, endosome-derived sEVs that are secreted by most cells,^12^ and are of interest to researchers because of their ideal native structures as nanocarriers,^13,14^ and their great potential for modulation and therapy.^12,15^ There is a characteristic subset of cell-derived components that are packaged in EVs, including proteins, nucleic acids, lipids, and glycoconjugates.^16^ Any components of EVs can be taken up by cells, may be involved in regulating the functions of target cells, and can be used for targeted therapy. For example, therapeutic miRNAs can be enriched in EVs to treat various diseases, including cardiovascular disease, musculoskeletal disease and cancer.^17^ In the GI tract, EVs are mainly secreted by immune cells and IECs,^18^ and are important contributors to intestinal homeostasis.^19^ Under inflammatory conditions, EVs can also participate in the regulation of the anti-inflammatory response, restoring mucosal barrier integrity and reconstituting the microbiota.^20^ In addition, another type of vesicles in GI is released from the microbiota, these are bacterial membrane vesicles (BMVs), which range in size from 20 to 400 nm in diameter, can be categorized based on their different parent cells, structure and composition, and include outer-MVs, outer-inner MVs from gram-negative bacteria and cytoplasmic MVs from gram-negative bacteria.^21^ Abundant evidence has proven that BMVs are involved in interactions between host and gut microbes and maintaining intestinal homeostasis.^22,23^

Currently, EVs-based treatment has developed as an advanced therapeutic approach for various diseases. However, the roles of vesicles from the host or the microbiota in the colon under inflammatory conditions remain unclear. In the present study, acute and chronic colitis rat models were established to investigate the changes in miRNA in EVs from colitis individuals and to evaluate the effects of these different miRNAs on colitis. Moreover, BMVs from fecal fermentation were administered to mice with colitis to investigate the roles of BMVs in the colon of mice. These results will establish a new EV-based interaction cycle between the host GI tract and gut microbiota in colitis conditions and form a theoretical basis for the treatment of colitis using EVs and BMVs.

## 2 Results

### 2.1 Differential expression of colonic EV-miRNAs in acute and remission of chronic colitis

**Figure 1.**
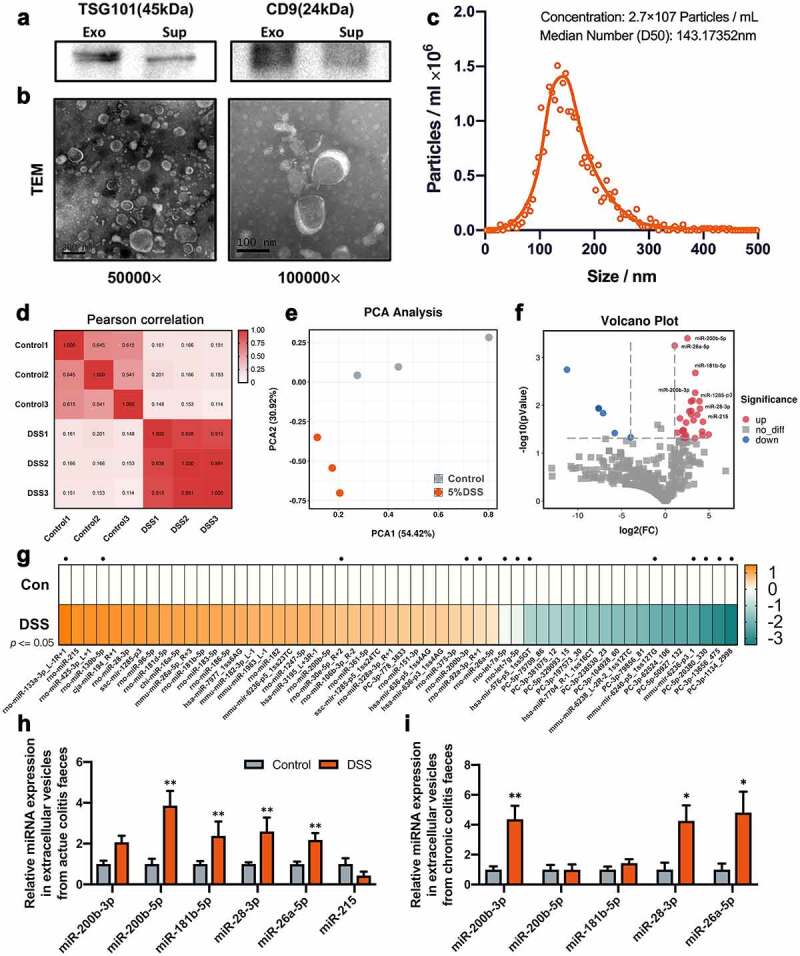
Difference of EVs-miRNA expression in acute and remission of chronic colitis. The identification of EVs from the colon using (a) immunoblot analysis, (b) Negative stain Transmission Electron Microscopy and (c) Nanoparticle Tracking Analysis. (d) The Pearson correlation, (e) principal component analysis (PCA) and (f) Volcano plot analysis of miRNA sequencing between Control and DSS groups. (g) Heatmap of relative miRNA expression according to miRNA sequence (p < .05), and black dots represent high expression in EVs. (h-i) Verification of different miRNA using RT-qPCR in acute and remission of chronic colitis models respectively (n = 7–8). Data were presented as the means ± SEM. * p < .05 vs. Control-DSS.

To investigate the changes in EV-miRNAs in colitis, acute colitis rat models were established using 7 days of DSS induction (Figure S1A). As previously described,^24^ the body weights and the length of colons in DSS-induced colitis rats decreased significantly (Figure S1B, S1D and S1E), and the DAI and colon damage index increased dramatically (Figure S1C, S1F and S1G). In addition, increased levels of LPS, TNF-α and IL-6 in the serum and aberrant mRNA and protein expression in the colon demonstrated that colon inflammation was severe, and the intestinal barrier was destroyed (Figure S1H-S1M). We isolated EVs from the colonic content of rats using gradient centrifugation and ultracentrifugation. EV markers were assayed by western blotting, and the sizes of EVs were observed by electron microscopy and nanoparticle tracking analysis for EV identification (Figure 1a-c). Subsequently, total RNA was isolated from EVs for miRNA sequencing. We found that the composition of miRNAs was significantly different between the control and DSS groups according to Pearson correlation, PCA and Volcano Plot analysis (Figure 1d-f). Importantly, several significant difference miRNAs were identified based on fold change, *p* value and initial abundance (Figure 1g), including miR-200b-3p, miR-200b-5p, miR-26a-5p, miR-215, miR-181b-5p and miR-28-3p, and verified using RT-qPCR (Figure 1h). The levels of miR-26a-3p, miR-200b-5p, miR-181b-5p and miR-28-3p increased significantly in the DSS group, according to the sequencing analysis results (Figure 1g). In addition, miR-200b-3p was highly expressed in the control (Figure 1g) and showed an increasing trend in the DSS group that was not significant (Figure 1h).

To determine the functions of differentially expressed miRNAs in colitis development, chronic colitis rat models were established using 3 intermittent exposures to 3% DSS (Figure S1N). Compared to those in the acute colitis model, the difference in body weights was not obvious during the first processing phase and gradually became more serious in the following phase (Figure S1O). The DAI score fluctuated during the period of exposure (Figure S1P). Two weeks of remission after the last DSS exposure, the length of the colon was still significantly decreased in the DSS group (Figure S1Q and S1R). However, the damage index of the colon in the DSS group decreased significantly when compared to the colon damage index in acute colitis (Figure S1S and S1T). The RT-qPCR and western blot data showed that the levels of mRNAs and protein associated with the gut barrier, including Claudin-1, Occludin and TJP-1, were significantly decreased (Figure S1V-X). However, the mRNA and protein levels of the cytokine IL-6 and TNF-α were stable (Figure S1U and S1V). Interestingly, the levels of Muc1 and Muc2 increased significantly during remission of chronic colitis (Figure S1V), indicating that the synthesis of epithelial mucin was activated during remission. We also found that the levels of miR-28-3p, miR-26a-5p and miR-200b-3p were significantly increased (Figure 1i). In particular, the fold change in the miR-200b-3p group was more notable than that in acute colitis (Figure 1h and i). In addition, the levels of miR-200b-5p and miR-181b-5p returned to normal levels (Figure 1i).

### 2.2 The abundance of miRNAs was significantly correlated with the colon bacterial community

**Figure 2.**
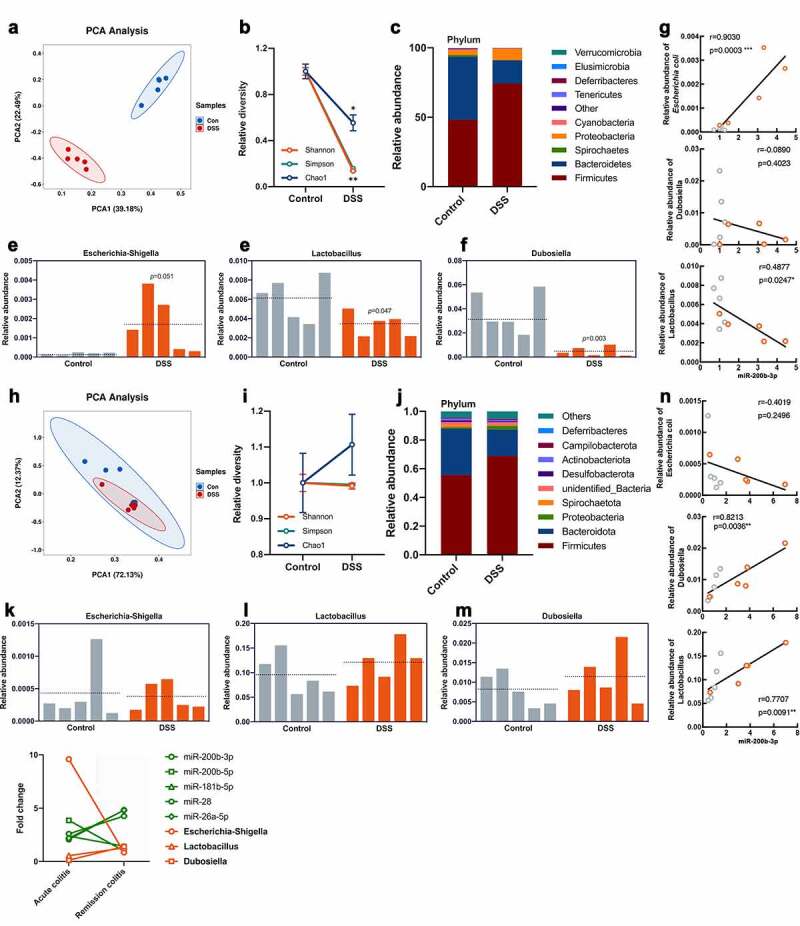
The correlation analysis of different miRNA and microbes. (a and k) PCA and (b and i) diversity of microbiota in acute and remission of chronic colitis. (B and I). (c and j) the relative abundance of microbiota in the colon at the phylum level. (D-F and K-M) the relative abundance of *Escherichia-Shigella, Lactobacillus* and *Dubosiella* in colitis. (g and n) Correlation analysis of the above three bacteria with miR-200b-3p. Values for *r* and *p* are indicated in each graph. (o) The fold change of bacteria and miRNA in acute and remission of chronic colitis.

The composition of the gut microbiota is an important factor in the development of colitis.^25^ Additionally, abundant evidence has indicated that miRNAs from the host shape the gut microbiota.^26^ Accordingly, we investigated the correlation between miRNAs and bacterial abundance in acute and remission of chronic colitis (Figure 2). In acute colitis, the composition of the microbiota in the DSS group was significantly different from that in the control group (Figure 2a), and there was decreased diversity (Figure 2b). At the phylum level, we found a shift in the dominant bacterial phyla Firmicutes and Bacteroidetes, as well as increased Proteobacteria (Figure 2c). We then constrained the relative abundance analysis to the three most common bacteria genera for 16S rRNA analysis: *Escherichia-Shigella, Lactobacillus* and *Dubosiella*. The increased abundance of *Escherichia-Shigella* in colitis has been reported repeatedly and was also observed in our experiments (Figure 2d). Furthermore, the levels of *Lactobacillus* and *Dubosiella* were significantly decreased in DSS individuals (Figure 2e and f). Combining the levels of EV-miRNAs and microbes in the colon, it was found that the miRNAs with increased levels were positively correlated with *Escherichia-Shigella* (Figure 2g and Figure S2A), except miR-200b-5p. In addition, *Dubosiella* and *Lactobacillus* were negatively correlated with these miRNAs, and the differences were especially significant for miR-200b-3p, miR-200b-5p and miR-26a-5p (Figure 2g and Figure S2A).

During a remission of chronic colitis, the phenotype of colitis was significantly relieved, and obvious restoration of the microbiota was observed compared to that in acute colitis. PCA and diversity analysis demonstrated that in terms of composition or diversity, there was little difference between the control and DSS groups (Figure 2h and 2i). Interestingly, the abundance of microbes at the phylum level was consistent with acute colitis (Figure 2j). However, three typical bacteria all showed a similar abundance to that in the control group (Figure 2k-m). Accordingly, the changes in microbes during the remission of chronic colitis were not associated with miRNA variations, except miR-200b-3p (Figure 2n and Figure S2B). The levels of miR-200b-3p showed a negative correlation with *Escherichia-Shigella* and a significantly positive correlation with *Dubosiella* and *Lactobacillus*, which was completely contrary to the results in acute colitis (Figure 2g). Combining the results of the acute and remission of chronic colitis models, we found an interesting phenomenon that the fold changes in miR-200b-3p, miR-26a-3p and miR-28 were increased from acute to remission in chronic colitis. The same changes were observed in *Dubosiella* and *Lactobacillus* but were sharply decreased in *Escherichia-Shigella* (Figure 2o), which indicated that along with the increasing abundance of these miRNAs, the levels of bacteria gradually decreased. While the abundance of miR-181-5p and miR-200b-5p did not show any correlation with bacteria in chronic colitis models. Therefore, we hypothesized that the abundance of some miRNAs was highly correlated with the colon bacterial community.

### 2.3 miRNA mimics transplantation restores colonic microbes and ameliorates DSS-induced colitis

**Figure 3.**
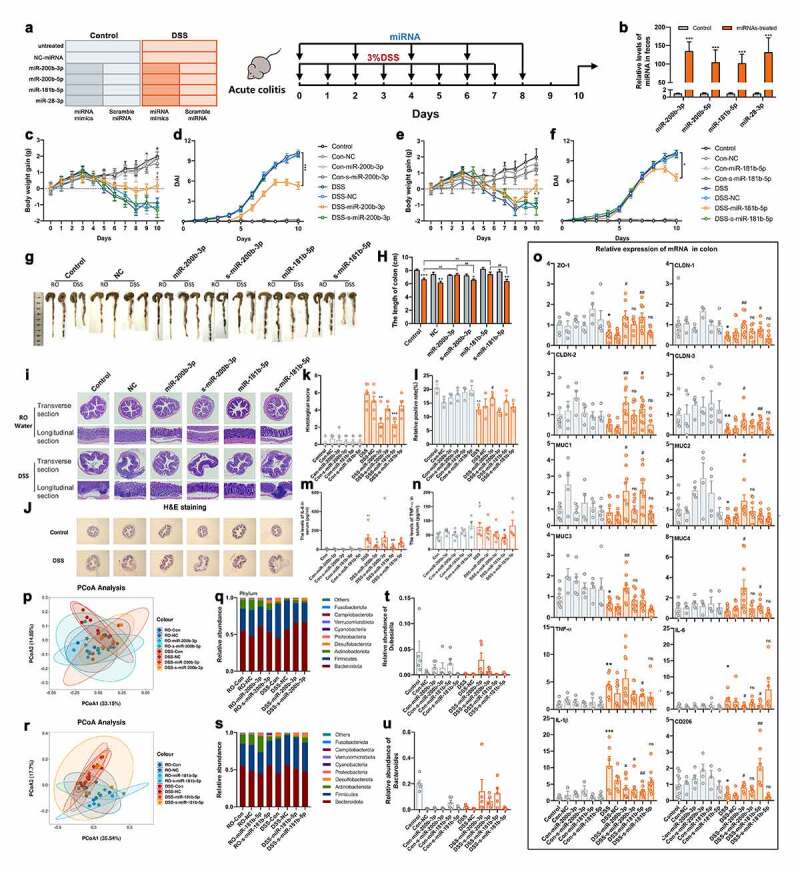
miR-200b-3p and miR-181b-5p transplantation restore DSS-induced colitis. (a) Mice treated with different miRNA mimics or scramble miRNA (10ug/mice) every other day at beginning of DSS exposure. (n = 10). (b) Relative levels of miRNAs in feces at 48 h after administration of miRNAs. (c and e) The body weight gain, (d and f) DAI score and (g and h) colon length of miR-200b-3p and miR-181b-5p treated mice. Data were presented as the means ± SEM. * p < .05, ** p < .01, vs. DSS; ^#^ p < .05, vs. s-miRNA groups. (i-l) H&E and AB-PAS staining of colons and quantitative analysis of inflammatory cells infiltration and mucus secretion. The levels of IL-6 (m) and TNF-α (n) in serum. Data were presented as the means ± SEM. * p < .05 vs. Control; ^#^ p < .05, ^##^ p < .01 vs. DSS. (o) the relative expression of mRNA related to intestinal barrier, mucin and inflammatory cytokines. Data were presented as the means ± SEM. * p < .05, ** p < .01, *** p < .001, vs. Control; ^#^ p < .05, ^##^ p < .01 vs. DSS. (p and r) UniFrac principal coordinate analysis (PCoA) and (q and s) relative abundance of microbiota in colonic content (n = 5). (t-u) the relative abundance of *Bacteroides* and *Dubosiella* in colonic content (n = 5). Data were presented as the means ± SEM. * p < .05, ** p < .01, vs. Control; ^#^ p < .05, vs. DSS.

To evaluate the role of these miRNAs in the development of colitis in vivo, 10 μg of miRNA mimics and scramble miRNA (s-miRNA), as a. negative control were directly administered to DSS-induced colitis mice by gavage every other day for 10 days (Figure 3a).^26^ The levels of miRNA in feces were detected after 48 h of miRNAs administration, the results showed that the content of miRNAs was significantly increased as previously describe (Figure 3b). We found that mice that were administrated miR-200b-3p and miR-181b-5p exhibited less bodyweight loss than mice in the DSS control group (Figure 3c and e), but no similar effects were observed in the DSS-s-miR-200b-3p and DSS-s-miR-181b-5p groups. Interestingly, compared to the effect of miR-200b-3p, rapid bodyweight gains from Day 8 were observed in the DSS-miR-181b-5p group (Figure 3e), suggesting there may be a lag phase in the effect of miR-181-5p. The DAI score also showed a mitigating effect in the DSS-miR-200b-3p and DSS-miR-181b-5p groups (Figure 3d and f). In addition, longer colon lengths and less severe colonic damage were also observed in these two groups (Figure 3h, i and k). Moreover, the results of Alcian blue-periodic acid Schiff (AB-PAS) staining showed that epithelial mucin was significantly destroyed in the DSS group and restored by miR-200b-3p and miR-181b-5p administration (Figure 3j and l). However, no similar effects were observed in miR-200-5p- and miR-28b-3p-treated colitis mice (Figure S3A-3j). Furthermore, the levels of IL-6 in serum were decreased in the DSS-miR-200b-3p and DSS-miR-181b-5p groups (Figure 3m), but decreased levels of TNF-α were only observed in the latter group (Figure 3n). The RT-qPCR results indicated that the mRNA expression of genes associated with barrier integrity, including ZO-1, the CLDN family, and the MUC family, decreased significantly in DSS-induced colitis, but were rescued in the colons of miR-200b-3p- or miR-181b-5p-treated mice (Figure 3o), especially the expression of CLDN1, CLDN2, MUC1 and MUC4. The decreased expression of proinflammatory cytokine and increased expression of anti-inflammatory cytokine were also observed in the miR-200b-3p- and miR-181b-5p-treated colitis groups, including IL-6, IL-1β and IL-10, but no significant changes in IL-22 was observed (Figure 3n and Figure S3K). Importantly, TNF-α decreased significantly in the miR-181b-5p treatment group but not in the DSS-miR-200b-3p group (Figure 3n). Moreover, the expression of CD206, which is a marker of M2 macrophages, significantly increased after miR-181b-5p treatment (Figure 3m), which demonstrated the effect on the DSS-miR-181b-5p group might be associated with macrophages polarization. In addition, a slight recovery was observed in the DSS-miR-200b-5p group, which was mainly associated with the expression of CLDN-1, CLDN-3, MUC2, and MUC4, but the effect was not obvious (Figure S3K). No difference was found in the miR-28-3p treatment group compared to the DSS and DSS-NC groups (Figure S3K and S3L).

Next, we investigated the difference in the microbiota in the miR-200b-3p- and miR-181b-5p-treated groups. The results showed that treatment with miR-200b-3p restored the DSS-induced changes in microbial composition (Figure 3o). At the phylum level, the shift in the dominant bacterial phyla that was previously decreased was reset in the DSS-miR-200b-3p group (Figure 3p). However, these differences were not significant in the miR-181b-5p-treated groups (Figure 3q and r). More importantly, at the genus level, the levels of *Bacteroides* and *Dubosiella* increased significantly in the DSS-miR-200b-3p group (Figure 3s and t).

### 2.4 miR-200b-3p directly affects the structure of the colonic bacterial community

**Figure 4.**
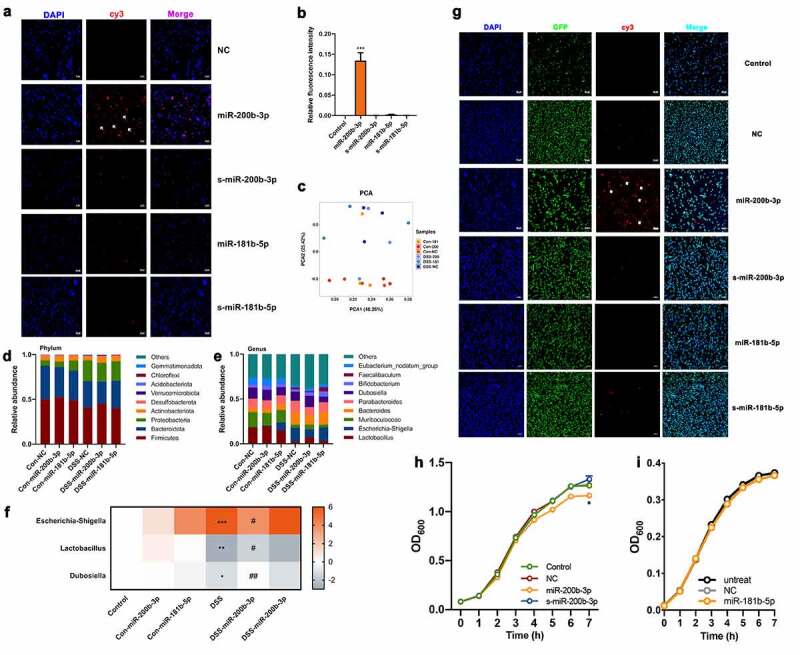
Effects of miR-200b-3p on microbiota and *E. coli*. (a-b) Representative confocal images of Cy3 (red) and DAPI (blue) for microbiota exposed to Cy3-labeled miRNA. Scale bar: 10 μm. Data were presented as the means ± SEM. (c) PCA and (d and e) relative abundance of microbiota. (f) the relative abundance of *Escherichia-Shigella, Lactobacillus* and *Dubosiella* in fecal fermentation. Data were presented as the means ± SEM. (g) Representative confocal images of GFP (green), Cy3 (red) and DAPI (blue) for *E. coli* GFP exposed to Cy3-labeled miR-200b-3p and Cy3- labeled miR-181b-5p. (h-i) The growth curve of predicted target genes of *E. coli*. Data were presented as the means ± SEM. * p < .05, vs. Control.

To further evaluate whether miRNAs can directly affect the abundance of microbes, batch in vitro fermentation models were used to evaluate the effects of miRNA. According to previous repetitive tests, microbial communities are very similar from day one to day six.^27^ Therefore, after 24 h in vitro fermentation, different synthesized fluorescently (Cy3)-conjugated miRNAs were added in the medium for 24 h and examined by confocal microscopy. We found that miR-200b-3p entered various bacteria and colocalized with bacterial nucleic acids, but there was no similar effect in miR-181b-5p-treated fermentation (Figure 4a and b). In addition, the 16S rRNA gene analysis of fermentation showed that there was a significant difference between the fermentation in the control and DSS groups, which could not be eliminated after miRNA exposure (Figure 4c). At the genus level, the abundance of *Lactobacillus* and *Dubosiella* was increased in the DSS-miR-200b-3p group, and the level of *Escherichia-Shigella* was decreased (Figure 4e and f). However, few changes were observed in the DSS-miR-181b-5p group (figure 4f).

Then, *Escherichia coli* (*E. coli*), which is a marker of colitis microbes, was selected to investigate the interactions with miRNA. *E. coli* GFP was cultured in LB medium at 37°C with 2 μM Cy3-labeled miR-200b-3p and miR-181b-5p. Confocal microscopy showed blue, green, and red fluorescence, which indicated nucleic acids, GFP and Cy3 labels, respectively (Figure 4g). Interestingly, miR-200-3p interacted with *E. coli* to affect growth (Figure 4g and h), but miR-181b-5p did not show any similar effects (Figure 4g and i). These results showed that miR-200b-3p could interact with some bacteria, just like *E. coli*, and in this way modulate microbiota composition.

### 2.5 BMVs from fecal fermentation exposed to miR-200b-3p ameliorated DSS-induced colitis

**Figure 5.**
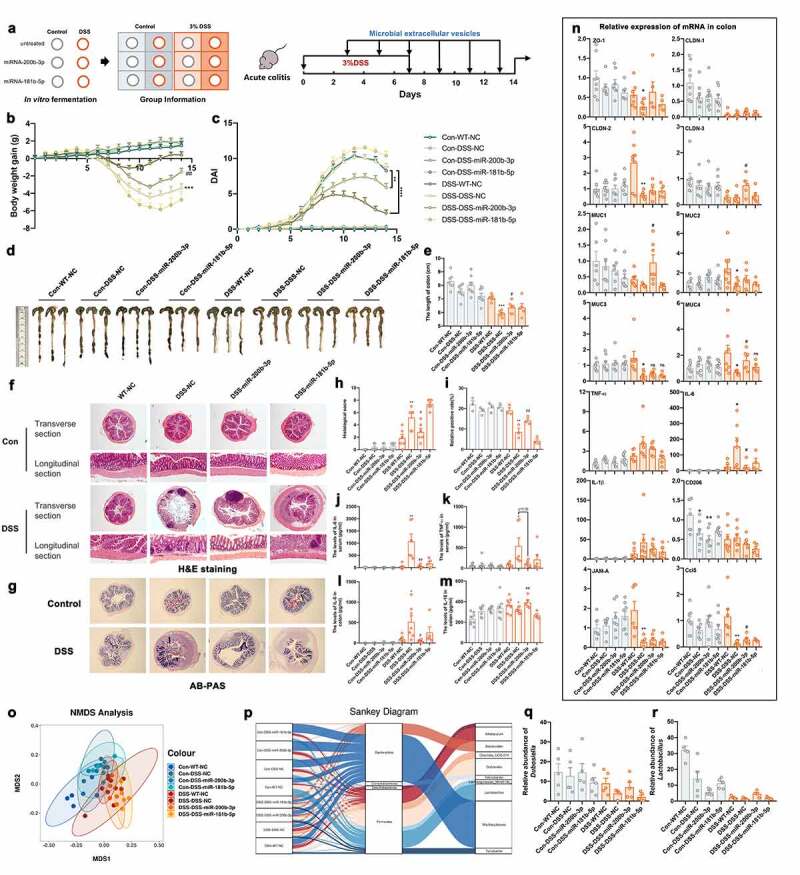
The effects of BMVs from miRNA exposure restore DSS-induced colitis. (a) Mice treated with BMVs (10ug/mice) from different miRNA exposure fermentation every other day for 5 times (n = 8). (b) The body weight gain, (c) DAI score and (d-e) colon length of BMVs treated mice. Data were presented as the means ± SEM. * p < .05, ** p < .01, *** p < .001, vs. DSS-WT-NC; ^#^ p < .05, ^##^ p < .01, vs. DSS-DS-NC. (j-m) the levels of IL-6, IL-10, TNF-α in serum and colon. Data were presented as the means ± SEM. * p < .05, ** p < .01, *** p < .001, vs. DSS-WT-NC; ^#^ p < .05, ^##^ p < .01, vs. DSS-DS-NC. (n) the expression of mRNA related to intestinal barrier, mucin and inflammatory cytokines. Data were presented as the means ± SEM. * p < .05, ** p < .01, vs. DSS-WT-NC; ^#^ *p* < .05, vs. DSS-DS-NC. (o) Nonmetric multidimensional scale (NMDS) and (p) Sankey analysis of microbiota in colonic content. (q-r) The relative abundance of *Lactobacillus* and *Dubosiella* in colonic content (n = 5). Data were presented as the means ± SEM.

The BMVs from microbes are also important regulators in the intestinal microenvironment according to previous evidence. Thus, we further evaluated the roles of BMVs from fecal fermentation products exposed to miRNA mimics in DSS-induced colitis. After 48 h of fermentation, the BMVs were isolated using gradient centrifugation and ultracentrifugation. Then, BMV solutions with equal amounts of protein were administered to DSS-induced colitis mice by gavage at a dose of 10 mg every other day (Figure 5a). Interestingly, the colitis groups treated with BMVs from the control and DSS-miR-200b-3p groups showed astonishing recovery compared to the DSS-DSS-NC group. Significant increases in body weight and decreases in DAI scores were observed (Figure 5b and c). Moreover, the lengths of the colons also increased in these two groups (Figure 5d and e). However, the group treated with BMVs from DSS-miR-181b-5p fermentation (DSS-DSS-mir-181b-5p) showed no recovery (Figure 5b-e), and an even higher mortality rate was observed in this group (results not shown). Normal mice administered BMVs from the DSS-miRNA groups (DSS-miR-200b-3p and DSS-miR-181b-5p) showed no effects on body weight (Figure 5b-e), colonic length or damage indices, and BMVs from normal feces treated with miRNAs did not rescue colitis (Figure S4A-S4D). According to H&E staining of the colon, in the DSS-DSS-NC group, two-thirds of the colon had lost its normal structure, and this effect was observed in colitis mice treated with BMVs from DSS-miR-181b-5p (Figure 5f and h). However, DSS-WT-NC and DSS-miR-200b-3p treatment resulted in colon morphology that was similar to that in the control group (Figure 5f and h). Correspondingly, similar results were observed by AB-PAS staining (Figure 5g and i). BMVs from normal feces exposed to miR-200b-3p and miR-181b-5p did not show any additional effects (Figure S4E -S4H).

In addition, colitis mice that were administered BMVs from normal and DSS-miR-200b-3p feces showed reduced levels of inflammation-related parameters in the serum and colon, including decreased IL-6 and TNF-α levels and increased IL-10 levels (Figure 5j-m). Similar results were observed in the mRNA levels of inflammatory cytokines, but no the effects on TNF-α and IL-1β were not significant (Figure 5n). The expression of genes associated with the epithelial barrier also significantly improved in these two groups, but the differences were not the same. In the DSS-WT-NC group, the expression of ZO-1, CLDN-2, MUC2, MUC3, and MUC4 was significantly increased, while CLDN-3, MUC1, and MUC4 were increased in the DSS-DSS-miR-200b-3p group (Figure 5n). These results demonstrated that the roles of BMVs from two different sources on colitis might be different.

According to NMDS analysis, in the MDS1-MDS2 quadrant, individuals in different groups were distributed from left to right (Figure 5o). The Con-WT-NC group occupied the leftmost position, while the DSS-DSS-NC and DSS-DSS-miR-181b-5p groups were distributed at the rightmost position, suggesting that there was a significant difference between the control and the latter two groups, but the DSS-WT-NC and DSS-DSS-miR-200b-3p groups showed higher similarity to Control (Figure 5o). Sankey Diagram showed that the level of *Dubosiella* was significantly decreased in DSS-induced colitis but rebounded in the DSS-WT-NC and DSS-DSS-miR-200b-3p groups (Figure 5p and q). In addition, the abundance of *Lactobacillus* was significantly decreased in the DSS-DSS-NC group and was partly restored in the DSS-DSS-miR-200b-3p group (Figure 5p and r).

### 2.6 miR-181b-5p targeted macrophages to relieve colitis

**Figure 6.**
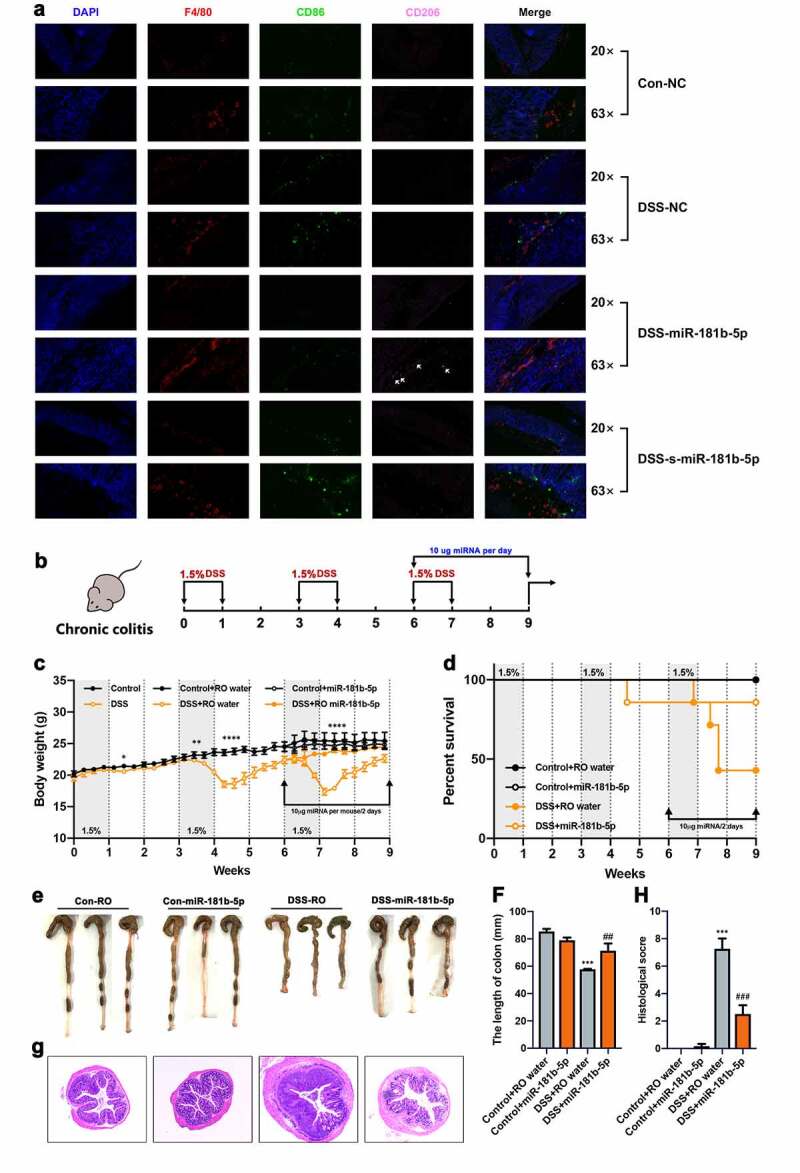
miR-181-5p restores DSS-induced colitis by targeting the polarization of macrophages. (a) Representative confocal images of F4/80 (red), DAPI (blue), CD86 (green) and CD206 (pink) for colon of mice treated with miR-181b-5p. (b) Chronic colitis mice were treated with miR-181b-5p mimics (10ug/mice) every other day at the last cycle of DSS exposure. (n = 8) (c) Body weight gain, (d) survival rate and (e-f) colon length of chronic colitis mice. Data were presented as the means ± SEM. *** p < .001, vs. Control; # p < .01, vs. DSS-RO. (g-h) H&E staining of colons and quantitative analysis of inflammatory cells infiltration. Data were presented as the means ± SEM. *** p < .001, vs. Control; ^###^ p < .001, vs. DSS-RO.

BMVs from the DSS-miR-181b-5p group did not ameliorate colitis, and an increase in CD206 was observed after miR-181-5p administration. Accordingly, we further investigated whether the roles of miR-181-5p on colitis were related to macrophage activation. Immunofluorescent staining was performed, and the red, green, and pink fluorescence represented total macrophages, antigen-presenting-associated cells and M2 macrophages, respectively. We found that the activity of antigen-presenting cells in the DSS-NC and DSS-s-miR-181b-5p groups was higher than that in the CON-NC and DSS-miR-181b-5p groups, which indicated that there was worse inflammation in the former two groups (Figure 6a). Moreover, a significant increase in the level of M2 macrophages was observed in the DSS-miR-181b-5p group compared to the other groups (Figure 6a). To further investigate the effects of miRNA on macrophages in vitro, Raw 264.7 cells were exposed to LPS (100 ng/ml) and combining treated with miRNA mimics, the results showed that miR-181b-5p partly inhibited LPS-induced M1 polarization (Figure S5). These results suggested that the effect of miR-181b-5p administration was dependent on M2 macrophage activation.

To verify the effects of miR-181b-5p on chronic colitis, chronic colitis mouse models were established using intermittent DSS exposure, and miR-181-5p was administered at the beginning of the last exposure cycle to simulate drug administration (Figure 6b). As expected, the pathology of chronic colitis was increasingly serious along with exposure cycles, affecting body weight, vitality and mortality (Figure 6c and d). On contrary, chronic colitis mice that were treated with miR-181b-5p showed significant reductions in body weight loss and mortality (Figure 6c and d). Furthermore, colon length was obvioulys increased (Figure 6e and f), and there were reductions in damage to the colonic epithelial barrier and inflammation (Figure 6g and h).

## 3 Discussion

Colitis is a widespread and complicated disease with no cure.^4^ In previous studies, researchers mainly investigated the effects of exogenous EVs on colitis, such as those from stem cells,^28^ plants, and milk.^29,30^ However, it is also important to investigate the changes of EVs and their components in the colon in different experimental models during the inflammation. Robust changes take place in both intestinal phenotype and bacterial structure when the normal intestine becomes inflamed, which specifically manifests as reductions in colon length and bacterial diversity,^31^ abnormal expression of epithelial barrier-related genes, and increased levels of inflammatory infiltration in the colon (Figure S1 and Figure 2).^32^ Under these conditions, active cell-to-cell interactions at the mucosal interface make the intestinal lumen a rich source of colitis-specific EVs.^33^ Additionally, the expression of mRNAs associated with inflammation, such as IL-6, IL-8 and TNF-α, was increased in EVs from IBD patients.^33^ Although several studies have investigated the miRNA expression profiles in IBD and the roles of differentially expressed miRNAs, such as miR-21,^34^ and miR-155,^35^ the changes in EV-miRNAs in colitis and the roles of host-microbe interactions remain unclear.

It is a challenge to obtain highly purified EVs. According to previous studies, ultracentrifugation is a widely accepted method for EV isolation.^36^ However, the composition of colonic content is complex and contains BMVs from microbes and RNAs associated with high-density lipoproteins. Therefore, a modified procedure was used in our study to remove most BMVs and bacterial debris from the sample.^37^ We found that the levels of several miRNAs in EVs from the colon were significantly increased in active acute colitis, and these miRNAs were increased in the colon or rectum in active IBD patients according to previous studies.^38,39^ In clinical therapy, UC is a lifelong disease with intermittent active phases. Accordingly, in the present study, intermittent DSS stimulation mimicked the clinical symptoms of colitis. In the remission between the two active phases of colitis, the abundance of miRNAs in EVs was different. Moreover, in this period, both the expression of mucosa-related mRNAs and levels of inflammatory infiltration were rescued, and intestinal symptoms and function were restored but accompanied by slight chronic inflammation. miRNAs are well-known mediators of cellular homeostasis,^40^ and are upregulated under stress-induced conditions, such as NF-κB activation in LPS-stimulated macrophages.^41^ In acute colitis models, EVs-miR-200b-3p was significantly increased, but in exogenous miRNA supplementation experiments, miR-200b-3p could significantly restore colonic inflammation symptoms. The detection time point of miRNA abundance in acute colitis is the third day after stopping DSS drinking water, which is also recognized as the most serious node of DSS-induced colitis. After this point, the inflammation begins to gradually subside, and the gut begins to enter a state of self-repair. It is a normal phenomenon that some miRNAs beneficial to intestinal ecological restoration increase at this node. Accordingly, we believed that some increased mRNAs in EVs are important regulators in gut self-repair during stress, and some miRNAs returned to normal through the feedback regulation of inflammatory response during remission (Figure 1).

Regarding the roles of miRNAs, we verified them in the mice colitis models according to the conservation of miRNA sequence in *Homo sapiens, Rattus norvegicus*, and *Mus musculus*. Specifically, The miR-181 family is highly conserved in different species^42^ and includes at least four mature miRNAs: miR-181a, miR-181b, miR-181c, and miR-181d. In a recent study, miR-181b inhibited M1 macrophage polarization and facilitated M2 polarization in coronary artery disease.^42^ In addition, exosomal-miR-181b significantly enhanced M2 polarization and inhibited inflammation by suppressing PRKCD and activating p-AKT.^43^ In the present study, we found that miR-181b-5p administration inhibited M1 macrophage polarization and promoted M2 polarization in both acute and chronic colitis, which effectively relieved the symptoms of colitis (Figure 3 and Figure 6).

As a member of the miR-200 family, miR-200b is generally recognized as one of the fundamental regulators of epithelial-mesenchymal transition (EMT) by targeting ZEB transcription factors,^44^ subsequently controlling the expression of a cluster of genes, including E-cadherin and vimentin. The expression of miR-200b is significantly decreased in cancer, including breast and lung adenocarcinoma,^45,46^ but increased in IBD.^39^ miR-200b-3p is the main existing mature miRNA of miR-200b according to miRbase (http://www.mirbase.org). Hence, in our present study, we initially thought that miR-200b-3p administration would rescue colitis by inhibiting the activation of EMT, which was proved by the reduced expression of the miR-200b target (The result is not displayed). However, a previous study has suggested that miRNAs can also directly enter bacteria, specifically regulate bacterial gene transcripts, and affect bacterial growth.^26^ Combined with changed microbiota in the miRNA administration experiment, these results supported the investigation of another mechanism by which miR-200b-3p alleviates colitis. Therefore, we investigated the effects of miR-200-3p on microbiota using batch in vitro fermentation models. Batch in vitro fermentations are the simplest methodology to simulate colonic fermentation.^27^ This model is typically used to study the interplay of a given compound with the gut microbes and has been reported in several studies.^47–49^ However, batch in vitro fermentations also have some limitations. The main issue is that this methodology is the farthest from physiological conditions,^27^ but suitable for our purposes. In addition, YCFA is a suitable medium for the growth of most bacteria and is often used to discover new intestinal anaerobic bacteria and simulate intestinal fermentation in vitro. Interestedly, we found that miR-200b-3p exposure shaped the gut microbiota composition in vivo and in vitro, including an increased abundance of *Lactobacillus, Dubosiella*, and decreased *Escherichia-Shigella*, which might be achieved by promoting or inhibiting the proliferation of certain bacteria, such as *E. coli* (Figure 4). However, the mechanism of how miR-200b-3p enters bacteria and affects the proliferation of bacteria could not be explained in this study and need more future studies. In general, These results suggest that miR-200b-3p can regulate gut homeostasis by interacting with gut microbes.

BMVs were thought to serve as a mechanism of intra‐kingdom communication to enable the transfer of bioactive molecules,^50,51^ which has been proven in recent studies.^23^ Due to the similar size and properties to EVs, the potential of BMVs for medical applications is also valued. The difference is that BMVs always load with some bacterial toxins and antigens, so they are often considered to be an excellent vaccine adjuvant.^52^ In abundant IBD-related studies, BMVs play an important role in the modulation of intestinal epithelial barrier integrity and immune response. Some commensal and probiotic-derived BMVs relieve colitis via reinforcing gut barrier integrity and activating immune cells.^53,54^ Our results also showed that BMVs were important regulators of host‐microbe communication. BMVs from normal mouse fecal fermentation could significantly change the microbial community and rescue experimental colitis. There is a stable microbial community with a high abundance of commensal and probiotics in a healthy gut. Therefore, BMVs isolated from normal gut microbiota reverse the composition of inflamed microbes in colitis via inhibition of pathogens. Similar effects were observed in the transplantation of BMVs from individual probiotics, but a more significant contribution to intestinal microecology was shown in the former. Additionally, BMVs from inflammatory feces exposed to miR-200b-3p showed similar alleviative effects on colitis. We have previously demonstrated that miR-200b-3p can reset the inflammatory microbes and inhibit the proliferation of pathogens, so that commensal bacteria can recover the dominant level and secrete more BMVs leading to a similar effect to the normal. These results well indicated that the effects of miR-200b-3p on colitis are partly dependent on microbes. Meanwhile, these results also well confirmed our hypothesis: EVs-miRNAs secreted in the colitic condition in mice can impact the composition of the bacterial community and reconstruct parent cells of the BMVs set, which can further affect the microecology in colitis. However, due to the inhibitory effect of miR-200b-3p on EMT, we did not confirm whether BMVs play a central role in the miR-200b-3p-mediated recovery of colitis, which was also a limitation of this study. According to our results, we thought that the effect of EVs-miR-200b-3p on bacterial BMVs was achieved by changing the abundance of different genera in the microbiota, whether it affects the secretion of bacterial BMVs and its mechanism needs more thorough studies. In addition, compared to the inflammatory intestine, the healthy intestine has a more stable microbiota with high diversity, which can explain why BMVs from normal feces exposed to miRNA showed little effect on colitis. In current clinical colitis treatment, FMT is an advanced therapy that uses feces reconstruction to repair disruption of the normal microbial communities. However, integral microbiota transplantation has an increased risk in IBD patients,^55^ as well as scarceness of excellent donors, limitation of storage and transfer. In addition, a single probiotic transplant may involve causing sepsis. Compared with integral microbiota transplantation, MV transplantation is more controllable and less risky and might be an easily available and sustainable therapy alternative to FMT.^20^

*Dubosiella* are anaerobic Gram-stain-positive bacteria that produce short-chain fatty acids (SCFAs).^56,57^ In previous studies, the abundance of *Dubosiella* was significantly decreased in DSS-induced colitis mice and increased during remission of colitis,^58,59^ which indicated that this bacterium is an important bacteria associated with the development and remission of colitis. In addition, *D. newyorkensis* is used as probiotics preparation for improving intestinal metabolism and immune ability, and antiaging of animals. In our present study, the alteration in the abundance of *Dubosiella* was observed in miR-200b-3p- and miR-181b-5p-treated colitis groups, as well as in MV-treated groups. In addition, *Dubosiella* was negatively correlated with the mRNA expression of IL-6 and TNF-α as previously described.^60^ However, studies on the effect and mechanism of *Dubosiella* in colitis are not available, and further studies are needed.

In summary, our study demonstrated the changes of EV-miRNAs in the inflamed colon and the roles of miR-200b-3p and miR-181b-5p in colitis. miR-181b-5p could target and regulate the polarization of intestinal macrophages to ameliorate colitis. The beneficial effects of miR-200b-3p on colitis were attributable in part to the reversion of the gut microbiota. Notably, BMVs also participate in the regulation of intestinal homeostasis. These results preliminarily build an EV-based host‐microbe interaction cycle in colitis (Figure S6). However, the specific mechanism by which EV-miRNAs and BMVs can treat colitis needs further investigation.

## 4 Experimental procedures

### 4.1 Animals

Animal procedures were approved by the Research Committee of Zhejiang University of Technology (Hangzhou, China) and the Zhejiang Academy of Agricultural Sciences (Hangzhou, China). The C57BL/6 J male mice (7–8 weeks) and Wistar male rats (7–8 weeks) were purchased from the China National Laboratory Animal Resource Center (Shanghai, China) and housed under specific pathogen-free (SPF) conditions with free access to water and commercial laboratory chow diet. Animals were used for experiments seven days thereafter. Tissues, serum and intestinal luminal contents were collected, snap-frozen and stored at −80°C for microbiota or miRNA analysis. Experiments were performed according to institutional guidelines and were approved by the Ethics Committee of the Zhejiang University of Technology (20201109134).

### 4.2 Models of Colitis

Several models of colitis were established by administering different doses of DSS (MW~40,000, Aladdin, Shanghai) in drinking water. The acute colitis mouse and rat models were induced by 3% and 5% DSS, respectively, for 7 consecutive days and DSS-free water for the next 3 days. For the chronic colitis models, 1.5% and 2.5% DSS were administered in drinking water for 7 days and then DSS-free water was administered for another 14 days. This drug delivery cycle was repeated twice. The disease activity index (DAI) was assessed daily in the acute colitis models and weekly in the chronic colitis models. The score was determined by assessing the following parameters: weight loss (0: none, 1: 1%−5%, 2: 5%−10%, 3: 10%−20%, 4: >20%); stool consistency (0 − 1: normal, 2 − 3: loose stool, 4: diarrhea); and fecal blood content (0 − 1: normal, 2: hemoccult positive, 4: hemorrhage/gross bleeding). Histologic grading of intestinal inflammation was defined by the infiltration of immunocytes (0: no change, 1: mild, 2: localized and moderate, 3: extensive and moderate, 4: extensive and severe), and the extent of epithelial/crypt damage (0, none; 1, basal 1/3; 2, basal 2/3; 3, crypt loss; 4, crypt and surface epithelial destruction).^61^

### 4.3 Histological analysis

Colonic tissues were collected, treated with 4% formalin and embedded in paraffin. Paraffin sections were stained with hematoxylin and eosin (H&E) and Alcian blue-periodic acid Schiff (AB-PAS). Immunohistochemical analysis with anti-F4/80 (GB113373, 1:3000), anti-CD206 (GB113397, 1:400), and anti-CD86 (GB13585, 1:3000) (Servicebio, Wuhan, China) were performed as described previously.

### 4.4 Cell culture and flow cytometry

The RAW 264.7 cells were obtained from Chinese Academy of Sciences, and cultured in complete high glucose DMEM (Gibco) which consists of 10% FBS (Gibco) and 1% penicillin as well as streptomycin. For studying macrophage polarization, RAW 264.7 cells were stimulated with 100 ng/ml LPS alone or combined with miRNA mimics at the indicated concentrations (2 μM) for 24 h and detected the surface markers CD86 and CD206 of RAW 264.7 using flow cytometry.

### 4.5 In vitro Fecal culture fermentation

Fresh fecal samples from the control and DSS groups were homogenized with 0.1 M anaerobic phosphate-buffered saline (PBS) and centrifuged at 1000 g for 20–30 s to remove undigested food particles. The suspension was centrifuged (8000 g) and washed twice in PBS before being resuspended in the same volume of PBS to make 10% (w/v) slurries. The growth media for in vitro fermentation was basic growth medium (YCFA) that was prepared as described previously and contained the following: 10 g/L tryptone, 2.5 g/L yeast extract, 1 g/L L-cysteine, 0.9 g/L NaCl, 0.09 g/L CaCl2 · 6 H2O, 0.45 g/L KH2PO4, 0.45 g/L K2HPO4, 0.09 g/L MgSO4 · 7H2O, 2 ml of vitamin I and hemin solution. The vitamin I solution contained the following components: 0.05 mg/ml vitamin B8, 0.05 mg/ml vitamin B12, 0.15 mg/ml acid 4-aminobenzoïque, 0.25 mg/ml vitamin B9 and 0.75 mg/ml pyridoxamine. The hemin concentration was 1 mg/ml in 1 M sodium hydroxide.^62^ Four milliliters of the fecal slurry were inoculated into 40 ml of growth medium and subjected to anaerobic fermentation under anaerobic conditions in an anaerobic workstation (80% N_2_, 10% CO_2_ and 10% H_2_). After 24 h of fermentation, miRNAs mimics were added and fermented for another 24 h. The precipitate and supernatant were collected for 16S rRNA gene analysis and extracellular vesicle (EV) isolation respectively.

### 4.6 Bacterial strain and growth measurements

*E. coli* (ATCC 29522) and *E. coli* GFP (DH5α GFP) were grown aerobically on NB medium for growth assays. *E. coli* was cultured in 5 ml aliquots of NB medium and grown aerobically to log phase. Subsequently, the cultures were inoculated (1/50) in 1 ml of fresh NB medium with 2 μM miRNAs.^26^ miRNA mimics and scramble miRNAs were synthesized by Sangon Biotech (Shanghai, China), and the sequences are listed in Table S2. Growth was monitored by measuring the absorbance at 600 nm (OD600) hourly for up to 7 hours with a spectrophotometer.

### 4.7 Confocal microscopy

*E. coli* GFP was aerobically cultured in LB medium at 37°C with shaking at 250 rpm shaking for 4 hours in the presence of 2 μM Cy3-labeled miRNAs (for the sequences, see Table S2). Fecal samples were anaerobically cultured in a YCFA medium at 37°C for 24 hours in the presence of 1 μM Cy3-labeled miRNAs.^26^ All bacteria were terminated with two washed of ice-cold PBS and were fixed with ice-cold 4% PFA. The samples were subjected to nucleic acid staining with DAPI and visualized with a 63× objective images captured with LSM 880 with Airyscan microscope systems (ZEISS, Germany). Images were processed with ImageJ software for channel merging, orthogonal views and reslicing.

### 4.8 EV isolation from colonic content

As previously description,^37^ the fresh colonic contents (1 g) were homogenized in 25 ml of ice-cold PBS, and centrifuged at 3000 × g for 10 min at 4°C, and this procedure was repeated twice. The supernatant was further centrifuged at 40,000 × g for 1.5 h at 4°C, and the supernatant was filtered through a 0.22 μm filter (Millipore) to remove microbes. Subsequently, the supernatant was centrifuged at 150,000 × g for 2.5 h at 4°C using an Optima L-100XP ultracentrifuge (Beckman, USA). The pellet was washed with 20 ml of ice-cold PBS and centrifuged at 150,000 x g for 10–15 mins at 4°C. Subsequently, the pellet was isolated using a sucrose density gradient ultracentrifugation for 3 h. Finally, the pellet was resuspended in 1 ml of PBS and stored at the 4°C fridges for subsequent analysis.

### 4.9 Bacterial EV isolation from in vitro fermented feces

After 48 h of fermentation at 37°C, the cells were removed from the supernatant by centrifugation (9000 × g, 15 min, 4°C), and the supernatant was filtered through a 0.22 μm filter (Millipore) to remove intact cells. The BMVs present in the supernatant was pelleted by subsequent ultracentrifugation (150,000 g, 4°C, 4 h) as previously described, washed with 20 ml of ice-cold PBS and ultracentrifuged (150,000 x g, 2 h, 4°C).^63^ The pellet was resuspended in 1 ml of PBS and protein concentration was determined using an Enhanced BCA Protein Assay Kit (Beyotime, Shanghai, China) according to the manufacturer’s instructions.

### 4.10 Negative stain transmission electron microscopy (TEM)

EVs and bacterial EVs were prepared for TEM by absorbing the samples (10) onto a 1–2 nm thick carbon film mounted on carbon-coated holey-film grids for 1 min. Following sample adsorption, the grids were quickly and gently blotted with filter paper, immediately floated for 5 min on 20 μl of 1% uranyl acetate and dried with filter paper. Imaging was performed on an HT7650 microscope (HITACHI, Japan).

### 4.11 Nanoparticle tracking analysis (NTA)

EV samples in particle-free PBS were analyzed by Multiple-Laser ZetaView PMX 110 Fluorescence Nanoparticle Tracking Analyzers (Particle MetriX, Germany) configured with a 488 nm laser and high sensitivity camera (0.703 μm/px) and were analyzed using ZetaView software (ZetaView 8.04.02 SP2). The typical concentration was approximately 5 × 10^7^ particles/ml for each measurement with 3 replicates.

### 4.12 Immunoblotting

Samples from EVs and colon were prepared in lithium dodecyl sulfate (LDS) sample buffer, and heated to 100°C for 10 min. The samples were separated by 8% or 12%

SDS-PAGE Bis-Tris gels (CoWin Bio, China) under nonreducing conditions, depending on the subsequent primary antibody used, before being transferred to Immobilon-FL PVDF Transfer Membranes (Millipore, USA). The PVDF membranes were blocked with TBS-T containing nonfat milk (5%) for 1 h and then blotted with primary antibodies against CD9, TSG101, Claudin-1 and Occludin (Abcam) overnight at 4°C. After being extensively washed with TBS-T, the membranes were further incubated with HRP-conjugated anti-rabbit IgG (Cell Signaling Technology) for 1 h at RT. Finally, the membranes were incubated with ECL detection reagents and visualized with a Luminescent Imaging Workstation (Tanon, China).

### 4.13 RNA extraction from the colon, EVs and E. coli, and quantitative real-time PCR

Total RNA was extracted from tissues and EVs as described previously. In brief, total RNA was extracted from all samples by RNA isolater Total RNA Extraction Reagent (Vazyme, China), and cDNA was synthesized using a reverse transcriptase kit (Vazyme, China). For tissue samples from the DSS group, the isolated RNA was purified using LiCl. In brief, a 0.1 volume of 8 M LiCl (Sigma-Aldrich) solution was added to a 1 volume of RNA solution and incubated on ice for 2 h before being centrifuged (14000 × g, 30 min, 4°C), after which the pellets were collected, and the process was repeated twice. Subsequently, 0.1 volume of 3 M sodium acetate (Thermo Scientific) and 2 volumes of ethanol were added to 1 volume of RNA solution to reprecipitate RNA without DSS. For EV samples, a settling agent (Takara #9094, Japan) was added with ethanol to extract the miRNAs. Subsequently, before reverse transcription, the RNA was processed with Poly(A) polymerase (NEBio, China) for the tailing reaction. The primers for miRNA reverse transcription were designed by miRprimer.^64^ Quantitative real-time PCR (RT-PCR) was performed as described previously.^65^ The primer sequences are shown in Table S1.

### 4.14 MicroRNA sequencing

Total RNA was prepared as described above. MicroRNA libraries were constructed using the Illumina TruSeq Small RNA Preparation Kit according to Illumina’s TruSeq Small RNA Sample Preparation Guide. The purified cDNA library was used for cluster generation on Illumina’s Cluster Station and then sequenced on an Illumina GAIIx, according to the vendor’s instructions. ACGT101-miR v4.2 (LC Sciences) was used for sequencing data analysis. Briefly, raw reads were subjected to the in-house program, ACGT101-miR, to remove adapter dimers, junk, low complexity results, common RNA families (rRNA, tRNA, snRNA, snoRNA), and repeats. Subsequently, unique sequences 18 to 26 bases in length were mapped to specific species precursors in miRBase 22.0 by BLAST search (Basic Local Alignment Search Tool) to identify known miRNAs and novel 3p- and 5p-derived miRNAs. The differentially expressed miRNAs identified based on normalized deep-sequencing counts were analyzed with the R package limma. The accession number for the miRNA sequencing data reports is NCBI Sequence Read Archive (SRA): PRJNA808643.

### 4.15 MicroRNA mimics and bacterial EVs transplantation

In the miRNA gavage experiment, miRNA mimics were dissolved in nuclease-free water and administered to DSS-induced colitis mice by gavage at a dose of 10 μg every other day for 10 days. Tissue and serum samples were collected on Day 10 after DSS treatment for pathological analysis. For the treatment of the chronic colitis model, miRNA mimics were administered at a dose of 10 µg every other day starting from the last cycle of DSS exposure until the end of the experiment.

For bacterial EV transplantation, after 48 h in vitro culture fermentation and exposure to miRNA mimics, EVs were collected from the supernatant and administered to DSS-induced colitis mice by gavage at three days after beginning DSS administration, at a dose of 10 µg every other day for 10 days. Tissue and serum samples were collected on Day 14 after DSS administration for pathological analysis.

### 4.16 Microbiome 16S rRNA gene analysis

Illumina HiSeq sequencing of the colonic microbiota was conducted (Novogene, Tianjin, China). The composition of the gut microbiome was determined by the Illumina HiSeq platform and QIIME2 bioinformatic analysis as previously described. Briefly, the CTAB/SDS method was used to extract the total genomic DNA from the samples. Then, 16S rRNA in distinct regions (16S V3-V4) was amplified with specific primers and barcodes and sequenced on an Illumina HiSeq platform. Quality filtering of the raw tags was performed using fastp (Version 0.20.0) software to obtain high-quality clean tags, which were annotated using QIIME2 software. The accession number for the 16S rRNA sequencing data reports is NCBI SRA: PRJNA808621. Finally, visualization was performed using the OmicStudio tools at https://www.omicstudio.cn/tool.

### 4.17 The levels of cytokine assay

Blood samples were centrifuged at 7000 rpm for 7 min, at 4°C, and then the supernatants were collected. Colonic tissues were weighed, homogenized, and centrifuged at 12,000 rpm for 15 min, at 4°C, and the supernatants were collected. The cytokine levels of the cytokines IL-10, IL-6, and TNF-α were quantified using a corresponding mouse ELISA kits (MULTISCIENCES, China) according to the manufacturer’s instructions.

### 4.18 Statistical analysis

All data were analyzed with GraphPad Prism 8.0 software and are presented as the means ± SEM. Differences between the mean values of the two groups were assessed using two-tailed Student’s t-tests. Correlation analyses were performed by Pearson’s correlation by using R statistical software (R version 3.5.3). Differences in mean values among more than two groups were determined using ANOVA, and p values < .05 were considered to indicate statistical significance.^66^

## Supplementary Material

Supplemental Material

## Data Availability

The miRNA and 16S rRNA sequencing data in this article are available in NCBI SRA. The accession numbers are PRJNA808643 (miR-seq) and PRJNA808621 (16S-seq).

## References

[1] McDowell C, Farooq U, Haseeb M. Inflammatory bowel disease. StatPearls. Treasure Island (FL): StatPearls Publishing Copyright © 2021, StatPearls Publishing LLC.; 2021. 29262182

[2] Kaplan GG. The global burden of IBD: from 2015 to 2025. Nat Rev Gastroenterol Hepatol. 2015;12:720–21. doi:10.1038/nrgastro.2015.150. 26323879

[3] Guan Q. A comprehensive review and update on the pathogenesis of inflammatory bowel disease. J Immunol Res. 2019;2019:7247238. doi:10.1155/2019/7247238. 31886308 PMC6914932

[4] Eisenstein M. Ulcerative colitis: towards remission. Nature. 2018;563:S33. doi:10.1038/d41586-018-07276-2. 30405234

[5] Lepage P, Colombet J, Marteau P, Sime-Ngando T, Dore J, Leclerc M. Dysbiosis in inflammatory bowel disease: a role for bacteriophages? Gut. 2008;57:424–425. doi:10.1136/gut.2007.134668. 18268057

[6] Morgan XC, Tickle TL, Sokol H, Gevers D, Devaney KL, Ward DV, Reyes JA, Shah SA, LeLeiko N, Snapper SB. Dysfunction of the intestinal microbiome in inflammatory bowel disease and treatment. Genome Biol. 2012;13:R79. doi:10.1186/gb-2012-13-9-r79. 23013615 PMC3506950

[7] Chang JT, Longo DL. Pathophysiology of inflammatory bowel diseases. N Engl J Med. 2020;383:2652–2664. doi:10.1056/NEJMra2002697. 33382932

[8] Abraham BP, Ahmed T, Ali T. Inflammatory bowel disease: pathophysiology and current therapeutic approaches. Handb Exp Pharmacol. 2017;239:115–146. doi:10.1007/164_2016_122. 28233184

[9] Boal Carvalho P, Cotter J. Mucosal healing in ulcerative colitis: a comprehensive review. Drugs. 2017;77:159–173. doi:10.1007/s40265-016-0676-y. 28078646

[10] Allegretti JR, Kelly CR, Grinspan A, Mullish BH, Hurtado J, Carrellas M, Marcus J, Marchesi JR, McDonald JAK, Gerardin Y, et al. Inflammatory bowel disease outcomes following fecal microbiota transplantation for recurrent C. difficile infection. Inflamm Bowel Dis. 2021;27:1371–1378. doi:10.1093/ibd/izaa283. 33155639 PMC8376126

[11] Pan BT, RM J. Fate of the transferrin receptor during maturation of sheep reticulocytes in vitro: selective externalization of the receptor. Cell. 1983;33:967–978. doi:10.1016/0092-8674(83)90040-5. 6307529

[12] Breakefield XO, Wood MJ, Breakefield XO, Wood MJA. Extracellular vesicles: biology and emerging therapeutic opportunities. Nat Rev Drug Discov. 2013;12:347–357. doi:10.1038/nrd3978. 23584393

[13] Bunggulawa EJ, Wang W, Yin T, Wang N, Durkan C, Wang Y, Wang G. Recent advancements in the use of exosomes as drug delivery systems. J Nanobiotechnol. 2018;16:81. doi:10.1186/s12951-018-0403-9. PMC6190562 30326899

[14] Luan X, Sansanaphongpricha K, Myers I, Chen H, Yuan H, Sun D. Engineering exosomes as refined biological nanoplatforms for drug delivery. Acta Pharmacol Sin. 2017;38:754–763. doi:10.1038/aps.2017.12. 28392567 PMC5520184

[15] Wiklander OPB, Brennan M, Lötvall J, Breakefield XO, El Andaloussi S. Advances in therapeutic applications of extracellular vesicles. Sci Transl Med. 2019;11:eaav8521. doi:10.1126/scitranslmed.aav8521. 31092696 PMC7104415

[16] Thery C, Zitvogel L, Amigorena S. Exosomes: composition, biogenesis and function. Nat Rev Immunol. 2002;2:569–579. doi:10.1038/nri855. 12154376

[17] Munir J, Yoon JK, Ryu S. Therapeutic miRNA-enriched extracellular vesicles: current approaches and future prospects. Cells. 2020;9:2271. doi:10.3390/cells9102271. PMC7601381 33050562

[18] van Niel G, Raposo G, Candalh C, Boussac M, Hershberg R, Cerf-Bensussan N, Heyman M. Intestinal epithelial cells secrete exosome-like vesicles. Gastroenterology. 2001;121:337–349. doi:10.1053/gast.2001.26263. 11487543

[19] Jiang L, Shen Y, Guo D, Yang D, Liu J, Fei X, Yang Y, Zhang B, Lin Z, Yang F, et al. EpCAM-dependent extracellular vesicles from intestinal epithelial cells maintain intestinal tract immune balance. Nat Commun. 2016;7:13045. doi:10.1038/ncomms13045. 27721471 PMC5062543

[20] Shen Q, Huang Z, Yao J, Jin Y. Extracellular vesicles-mediated interaction within intestinal microenvironment in inflammatory bowel disease. J Adv Res. 2021;37:221–233. doi:10.1016/j.jare.2021.07.002. 35499059 PMC9039646

[21] Toyofuku M, Nomura N, Eberl L. Types and origins of bacterial membrane vesicles. Nat Rev Microbiol. 2019;17:13–24. doi:10.1038/s41579-018-0112-2. 30397270

[22] Chu H, Khosravi A, Kusumawardhani IP, Kwon AHK, Vasconcelos AC, Cunha LD, Mayer AE, Shen Y, Wu W-L, Kambal A. Gene-microbiota interactions contribute to the pathogenesis of inflammatory bowel disease. Science. 2016;352:1116–1120. doi:10.1126/science.aad9948. 27230380 PMC4996125

[23] Bittel M, Reichert P, Sarfati I, Dressel A, Leikam S, Uderhardt S, Stolzer I, Phu TA, Ng M, Vu NK, et al. Visualizing transfer of microbial biomolecules by outer membrane vesicles in microbe-host-communication in vivo. J Extracell Vesicles. 2021;10:e12159. doi:10.1002/jev2.12159. 34664784 PMC8524437

[24] Ma L, Ni L, Yang T, Mao P, Huang X, Luo Y, Jiang Z, Hu L, Zhao Y, Fu Z, et al. Preventive and therapeutic spermidine treatment attenuates acute colitis in mice. J Agric Food Chem. 2021;69:1864–1876. doi:10.1021/acs.jafc.0c07095. 33541082

[25] Huttenhower C, Kostic AD, Xavier RJ. Inflammatory bowel disease as a model for translating the microbiome. Immunity. 2014;40:843–854. doi:10.1016/j.immuni.2014.05.013. 24950204 PMC4135443

[26] Liu S, da Cunha AP, Rezende RM, Cialic R, Wei Z, Bry L, Comstock L, Gandhi R, Weiner H. The host shapes the gut microbiota via fecal MicroRNA. Cell Host Microbe. 2016;19:32–43. doi:10.1016/j.chom.2015.12.005. 26764595 PMC4847146

[27] Perez-Burillo S, Molino S, Navajas-Porras B, Valverde-Moya AJ, Hinojosa-Nogueira D, Lopez-Maldonado A, Pastoriza S, Rufián-Henares JÁ. An in vitro batch fermentation protocol for studying the contribution of food to gut microbiota composition and functionality. Nat Protoc. 2021;16:3186–3209. doi:10.1038/s41596-021-00537-x. 34089022

[28] Heidari N, Abbasi-Kenarsari H, Namaki S, Baghaei K, Zali MR, Ghaffari Khaligh S, Hashemi SM. Adipose-derived mesenchymal stem cell-secreted exosome alleviates dextran sulfate sodium-induced acute colitis by Treg cell induction and inflammatory cytokine reduction. J Cell Physiol. 2021;236:5906. doi:10.1002/jcp.30275. 33728664

[29] Liao Y, Du X, Li J, Lonnerdal B. Human milk exosomes and their microRNAs survive digestion in vitro and are taken up by human intestinal cells. Mol Nutr Food Res. 2017;61:1700082. doi:10.1002/mnfr.201700082. 28688106

[30] Teng Y, Ren Y, Sayed M, Hu X, Lei C, Kumar A, Hutchins E, Mu J, Deng Z, Luo C, et al. Plant-derived exosomal MicroRNAs shape the gut microbiota. Cell Host Microbe. 2018;24:637–52 e8. doi:10.1016/j.chom.2018.10.001. 30449315 PMC6746408

[31] Franzosa EA, Sirota-Madi A, Avila-Pacheco J, Fornelos N, Haiser HJ, Reinker S, Vatanen T, Hall AB, Mallick H, McIver LJ, et al. Gut microbiome structure and metabolic activity in inflammatory bowel disease. Nat Microbiol. 2019;4:293–305. doi:10.1038/s41564-018-0306-4. 30531976 PMC6342642

[32] Mosli MH, Feagan BG, Zou G, Sandborn WJ, D’Haens G, Khanna R, Behling C, Kaplan K, Driman DK, Shackelton LM, et al. Reproducibility of histological assessments of disease activity in UC. Gut. 2015;64:1765–1773. doi:10.1136/gutjnl-2014-307536. 25360036

[33] Mitsuhashi S, Feldbrugge L, Csizmadia E, Mitsuhashi M, Robson SC, Moss AC. Luminal Extracellular Vesicles (EVs) in Inflammatory Bowel Disease (IBD) exhibit proinflammatory effects on epithelial cells and macrophages. Inflamm Bowel Dis. 2016;22:1587–1595. doi:10.1097/MIB.0000000000000840. 27271497 PMC4911338

[34] Ando Y, Mazzurana L, Forkel M, Okazaki K, Aoi M, Schmidt PT, Mjösberg J, Bresso F. Downregulation of MicroRNA-21 in colonic CD3+ T cells in UC remission. Inflamm Bowel Dis. 2016;22:2788–2793. doi:10.1097/MIB.0000000000000969. 27824649

[35] Wei M, Gao X, Liu L, Li Z, Wan Z, Dong Y, Chen X, Niu Y, Zhang J, Yang G, et al. Visceral adipose tissue derived exosomes exacerbate colitis severity via pro-inflammatory MiRNAs in high fat diet fed mice. ACS Nano. 2020;14:5099–5110. doi:10.1021/acsnano.0c01860. 32275391

[36] Thery C, Witwer KW, Aikawa E, Alcaraz MJ, Anderson JD, Andriantsitohaina R, Antoniou A, Arab T, Archer F, Atkin-Smith GK, et al. Minimal information for studies of extracellular vesicles 2018 (MISEV2018): a position statement of the International Society for Extracellular Vesicles and update of the MISEV2014 guidelines. J Extracell Vesicles. 2018;7:1535750. doi:10.1080/20013078.2018.1535750. 30637094 PMC6322352

[37] Yang C, Zhang M, Sung J, Wang L, Jung Y, Merlin D. Isolation and characterization of exosomes from mouse feces. Bio Protoc. 2020;10:e3584. doi:10.21769/BioProtoc.3584. PMC7241525 32440530

[38] Fasseu M, Treton X, Guichard C, Pedruzzi E, Cazals-Hatem D, Richard C, Aparicio T, Daniel F, Soulé J-C, Moreau R, et al. Identification of restricted subsets of mature microRNA abnormally expressed in inactive colonic mucosa of patients with inflammatory bowel disease. PLoS One. 2010;5:e13160. doi:10.1371/journal.pone.0013160. 20957151 PMC2950152

[39] Wu F, Guo NJ, Tian H, Marohn M, Gearhart S, Bayless TM, Brant SR, Kwon JH. Peripheral blood microRNAs distinguish active ulcerative colitis and Crohn’s disease. Inflamm Bowel Dis. 2011;17:241–250. doi:10.1002/ibd.21450. 20812331 PMC2998576

[40] Olejniczak M, Kotowska-Zimmer A, Krzyzosiak W. Stress-induced changes in miRNA biogenesis and functioning. Cell Mol Life Sci. 2018;75:177–191. doi:10.1007/s00018-017-2591-0. 28717872 PMC5756259

[41] Ma X, Becker Buscaglia LE, Barker JR, Li Y. MicroRNAs in NF-kappaB signaling. J Mol Cell Biol. 2011;3:159–166. doi:10.1093/jmcb/mjr007. 21502305 PMC3104013

[42] Yang Z, Wan X, Gu Z, Zhang H, Yang X, He L, Miao R, Zhong Y, Zhao H. Evolution of the mir-181 microRNA family. Comput Biol Med. 2014;52:82–87. doi:10.1016/j.compbiomed.2014.06.004. 25016292

[43] Liu W, Yu M, Chen F, Wang L, Ye C, Chen Q, Zhu Q, Xie D, Shao M, Yang L, et al. A novel delivery nanobiotechnology: engineered miR-181b exosomes improved osteointegration by regulating macrophage polarization. J Nanobiotechnol. 2021;19:269. doi:10.1186/s12951-021-01015-y. PMC8424816 34493305

[44] Korpal M, Lee ES, Hu G, Kang Y. The miR-200 family inhibits epithelial-mesenchymal transition and cancer cell migration by direct targeting of E-cadherin transcriptional repressors ZEB1 and ZEB2. J Biol Chem. 2008;283:14910–14914. doi:10.1074/jbc.C800074200. 18411277 PMC3258899

[45] Pogribny IP, Filkowski JN, Tryndyak VP, Golubov A, Shpyleva SI, Kovalchuk O. Alterations of microRNAs and their targets are associated with acquired resistance of MCF-7 breast cancer cells to cisplatin. Int J Cancer. 2010;127:1785–1794. doi:10.1002/ijc.25191. 20099276

[46] Rui W, Bing F, Hai-Zhu S, Wei D, Long-Bang C. Identification of microRNA profiles in docetaxel-resistant human non-small cell lung carcinoma cells (SPC-A1). J Cell Mol Med. 2010;14:206–214. doi:10.1111/j.1582-4934.2009.00964.x. 19900214 PMC3837589

[47] Nissen L, Casciano F, Gianotti A. Intestinal fermentation in vitro models to study food-induced gut microbiota shift: an updated review. FEMS Microbiol Lett. 2020;367:fnaa097. doi:10.1093/femsle/fnaa097. 32510557

[48] Li Z, Li Z, Zhu L, Dai N, Sun G, Peng L, Wang X, Yang Y. Effects of Xylo-Oligosaccharide on the gut microbiota of patients with ulcerative colitis in clinical remission. Front Nutr. 2021;8:778542. doi:10.3389/fnut.2021.778542. 35028306 PMC8748261

[49] Zhang Q, Zhao W, Zhao Y, Duan S, Liu WH, Zhang C, Sun S, Wang T, Wang X, Hung W-L, et al. In vitro study of bifidobacterium lactis BL-99 with fructooligosaccharide synbiotics effected on the intestinal microbiota. Front Nutr. 2022;9:890316. doi:10.3389/fnut.2022.890316. 35571919 PMC9096902

[50] Blenkiron C, Simonov D, Muthukaruppan A, Tsai P, Dauros P, Green S, Hong J, Print CG, Swift S, Phillips AR, et al. Uropathogenic *Escherichia coli* releases extracellular vesicles that are associated with RNA. PLoS One. 2016;11:e0160440. doi:10.1371/journal.pone.0160440. 27500956 PMC4976981

[51] Koeppen K, Hampton TH, Jarek M, Scharfe M, Gerber SA, Mielcarz DW, Demers EG, Dolben EL, Hammond JH, Hogan DA, et al. A novel mechanism of host-pathogen interaction through sRNA in bacterial outer membrane vesicles. PLoS Pathog. 2016;12:e1005672. doi:10.1371/journal.ppat.1005672. 27295279 PMC4905634

[52] Shen Q, Xu B, Wang C, Xiao Y, Jin Y. Bacterial membrane vesicles in inflammatory bowel disease. Life Sci. 2022;306:120803. doi:10.1016/j.lfs.2022.120803. 35850249

[53] Diaz-Garrido N, Badia J, Baldoma L. Microbiota-derived extracellular vesicles in interkingdom communication in the gut. J Extracell Vesicles. 2021;10:e12161. doi:10.1002/jev2.12161. 34738337 PMC8568775

[54] Ma L, Shen Q, Lyu W, Lv L, Wang W, Yu M, Yang H, Tao S, Xiao Y. *Clostridium butyricum* and its derived extracellular vesicles modulate gut homeostasis and ameliorate acute experimental colitis. Microbiol Spectr. 2022;10:e0136822. doi:10.1128/spectrum.01368-22. 35762770 PMC9431305

[55] Dailey FE, Turse EP, Daglilar E, Tahan V. The dirty aspects of fecal microbiota transplantation: a review of its adverse effects and complications. Curr Opin Pharmacol. 2019;49:29–33. doi:10.1016/j.coph.2019.04.008. 31103793

[56] Cox LM, Sohn J, Tyrrell KL, Citron DM, Lawson PA, Patel NB, Iizumi T, Perez-Perez GI, Goldstein EJC, Blaser MJ, et al. Description of two novel members of the family Erysipelotrichaceae: ilei*bacterium v*alens gen. nov., sp. nov. and Dubosiella newyorkensis, gen. nov., sp. nov., from the murine intestine, and emendation to the description of Faecalibaculum rodentium. Int J Syst Evol Microbiol. 2017;67:1247–1254. doi:10.1099/ijsem.0.001793. 28100298 PMC5817276

[57] Bojović K, Ignjatović ÐI, Soković Bajić S, Vojnović Milutinović D, Tomić M, Golić N, Tolinački M. Gut microbiota dysbiosis associated with altered production of short chain fatty acids in children with neurodevelopmental disorders. Front Cell Infect Microbiol. 2020;10:223. doi:10.3389/fcimb.2020.00223. 32509596 PMC7248180

[58] Wan F, Zhong R, Wang M, Zhou Y, Chen Y, Yi B, Hou F, Liu L, Zhao Y, Chen L, et al. Caffeic acid supplement alleviates colonic inflammation and oxidative stress potentially through improved gut microbiota community in mice. Front Microbiol. 2021;12:784211. doi:10.3389/fmicb.2021.784211. 34867926 PMC8636926

[59] Zhai Z, Zhang F, Cao R, Ni X, Xin Z, Deng J, Wu G, Ren W, Yin Y, Deng B, et al. Cecropin A alleviates inflammation through modulating the gut microbiota of C57BL/6 mice with DSS-induced IBD. Front Microbiol. 2019;10:1595. doi:10.3389/fmicb.2019.01595. 31354682 PMC6635700

[60] Wan F, Han H, Zhong R, Wang M, Tang S, Zhang S, Hou F, Yi B, Zhang H. Dihydroquercetin supplement alleviates colonic inflammation potentially through improved gut microbiota community in mice. Food Funct. 2021;12:11420–11434. doi:10.1039/D1FO01422F. 34673859

[61] Kitamoto S, Nagao-Kitamoto H, Jiao Y, Gillilland MG, Hayashi A, Imai J, Sugihara K, Miyoshi M, Brazil JC, Kuffa P, et al. The intermucosal connection between the mouth and gut in commensal pathobiont-driven colitis. Cell. 2020;182:447–62 e14. doi:10.1016/j.cell.2020.05.048. 32758418 PMC7414097

[62] Chen J, Pi X, Liu W, Ding Q, Wang X, Jia W, Zhu L. Age-related changes of microbiota in midlife associated with reduced saccharolytic potential: an in vitro study. BMC Microbiol. 2021;21:47. doi:10.1186/s12866-021-02103-7. 33588748 PMC7885556

[63] Schild S, Nelson EJ, Bishop AL, Camilli A. Characterization of Vibrio cholerae outer membrane vesicles as a candidate vaccine for cholera. Infect Immun. 2009;77:472–484. doi:10.1128/IAI.01139-08. 19001078 PMC2612262

[64] Busk PK. A tool for design of primers for microRNA-specific quantitative RT-qPCR. BMC Bioinform. 2014;15:29. doi:10.1186/1471-2105-15-29. PMC3922658 24472427

[65] Xie X, Shen Q, Yu C, Xiao Q, Zhou J, Xiong Z, Li Z, Fu Z. Depression-like behaviors are accompanied by disrupted mitochondrial energy metabolism in chronic corticosterone-induced mice. J Steroid Biochem Mol Biol. 2020;200:105607. doi:10.1016/j.jsbmb.2020.105607. 32045672

[66] Ma L, Ni Y, Wang Z, Tu W, Ni L, Zhuge F, Zheng A, Hu L, Zhao Y, Zheng L, et al. Spermidine improves gut barrier integrity and gut microbiota function in diet-induced obese mice. Gut Microbes. 2020;12:1–19. doi:10.1080/19490976.2020.1832857. PMC7668533 33151120

