# Supplementary material for: Extracellular vesicle miRNAs promote the intestinal microenvironment by interacting with microbes in colitis
Source: Gut Microbes. 2022 Sep 29;14(1):2128604. doi: 10.1080/19490976.2022.2128604 (PMC9542864; doi:10.1080/19490976.2022.2128604)
Supplement: Supplemental Material [file KGMI_A_2128604_SM7725.pdf]

Supporting Information

Extracellular vesicle miRNAs promote the intestinal microenvironment by interacting with microbes in colitis

Qichen Shen, Zhuizui Huang, Lingyan Ma, Jiachen Yao, Ting Luo, Yao Zhao, Yingping Xiao*, Yuanxiang Jin*

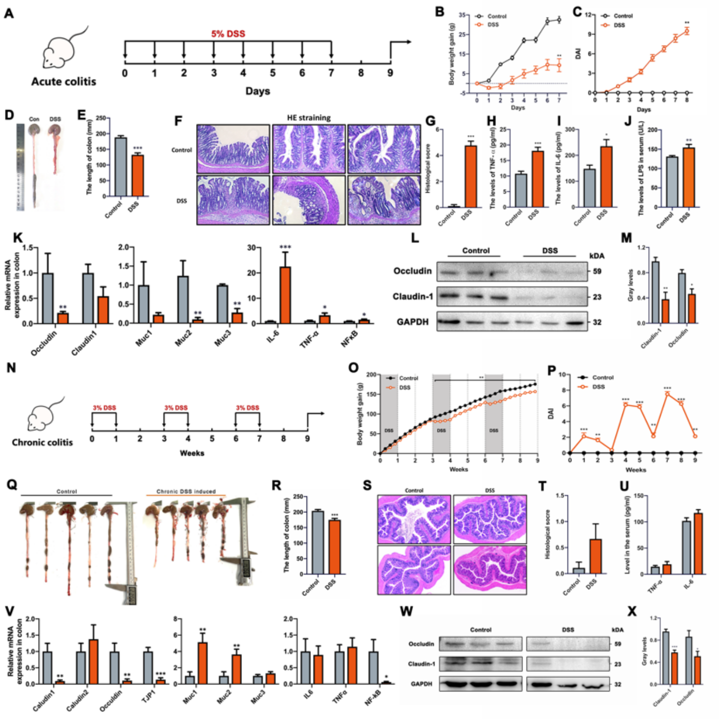

Figure S1 Phenotype of acute and chronic colitis rat models.
(A) Acute colitis rat models were established using 7 days of DSS induction (n = 15). (B and O) The body weight gain, (C and P) DAI score and (D, E, Q and R) colon length of colitis rats. Data were presented as the means ± SEM. * p < .05, ** p < .01, *** p < .001, vs. Control. (F, G, S and T) H&E staining of colons and quantitative analysis of inflammatory cells infiltration. (H-J and U) The levels of IL-6, TNF-α and LPS in serum. Data were presented as the means ± SEM. * p < .05, ** p < .01, *** p < .001, vs. Control. (K and V) The relative expression of mRNA related to intestinal barrier, mucin and inflammatory cytokines. Data were presented as the means ± SEM. * p < .05, ** p < .01, *** p < .001, vs. Control. (L, M, W and X) The results and quantification of western blot. Data were presented as the means ± SEM. * p < .05, ** p < .01, *** p < .001, vs. Control. (N) Chronic colitis rat models were established using 3 intermittent exposures to 3% DSS (n = 12).

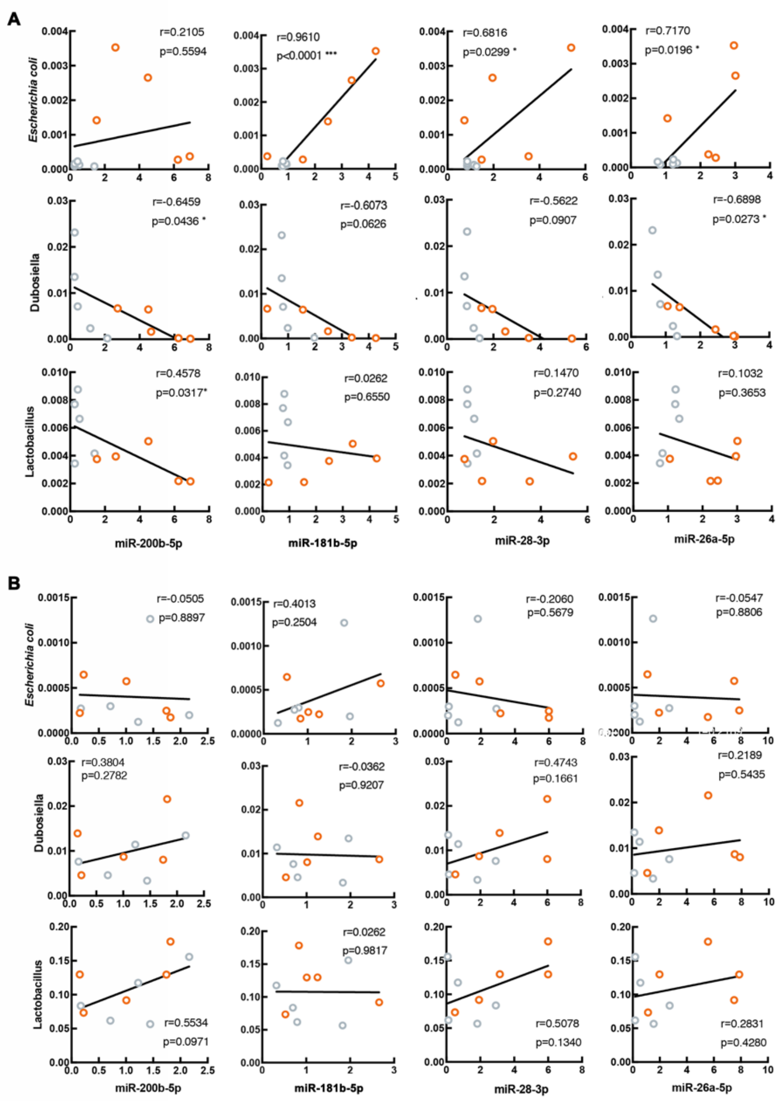

Figure S2 Correlation analysis of bacteria.

(A and B) Correlation analysis of *Escherichia-Shigella*, *Lactobacillus* and *Dubosiella* with miR-181b-5p, miR-200b-5p, miR-28-3p and miR-26a-5p. Values for *r* and *p* are indicated in each graph.

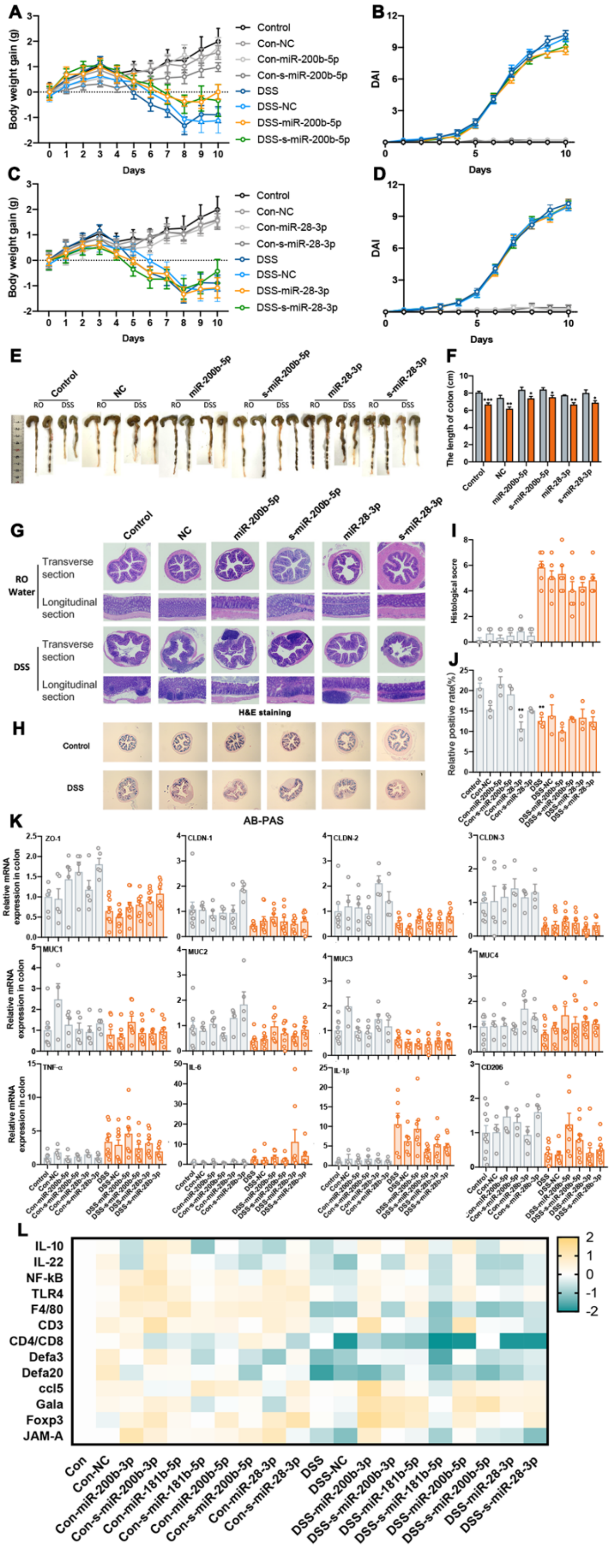

Figure S3 Effects of miR-200b-5p and miR-28-3p transplantation on colitis.

(A and C) The body weight gain, (B and D) DAI score and (E and F) colon length of miR-200b-5p and miR-28-3p treated mice. Data were presented as the means ± SEM. * $p < .05$, ** $p < .01$, *** $p < .001$, vs. Control. (G-J) H&E and AB-PAS staining of colons and quantitative analysis of inflammatory cells infiltration and mucus secretion. (K-L) The relative expression of mRNA

related to intestinal barrier, mucin and inflammatory cytokines.

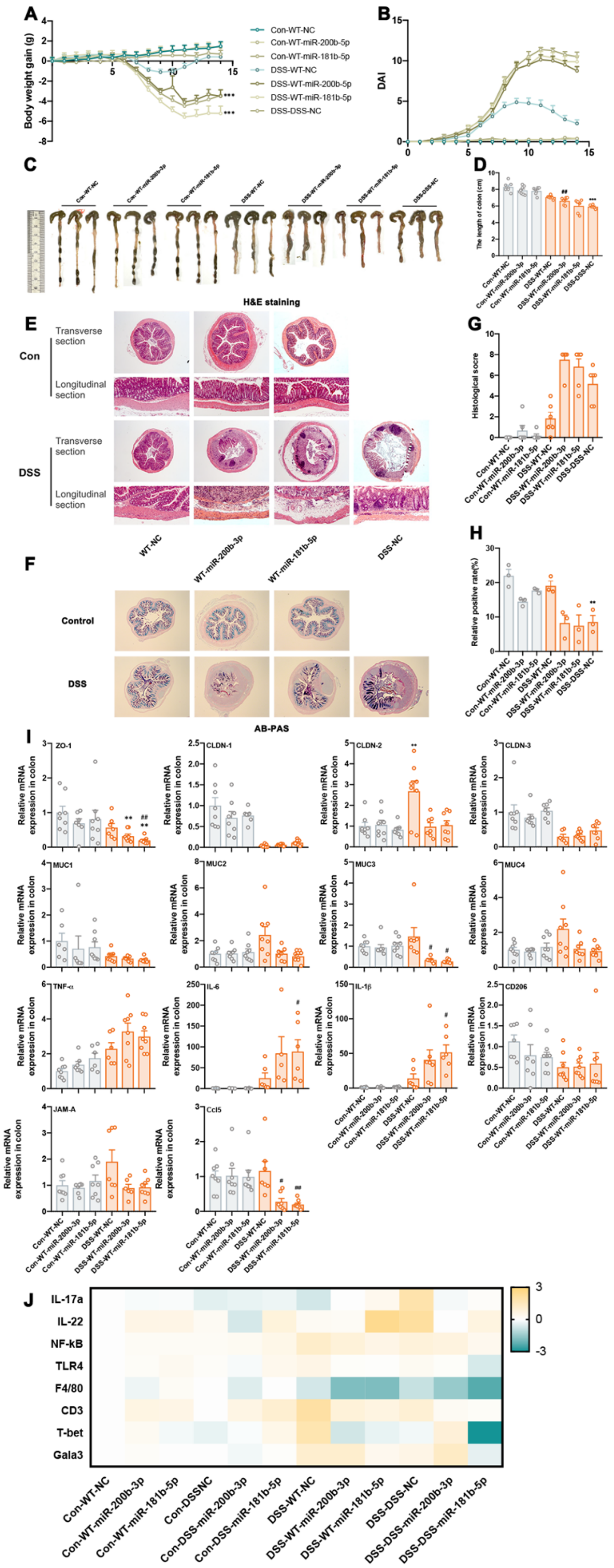

Figure S4 Effects of MVs from microbiota under miR-181b-5p exposure on colitis.

(A) The body weight gain, (B) DAI score and (C-D) colon length of MVs treated mice. Data were presented as the means ± SEM. *** $p < .001$, vs. DSS-WT-NC; ## $p < 0.01$, vs. DSS-DS-NC. (E-H) H&E and AB-PAS staining of colons and quantitative analysis of inflammatory cells infiltration and mucus secretion. (I and J) The relative expression of mRNA related to intestinal barrier, mucin and inflammatory cytokines.

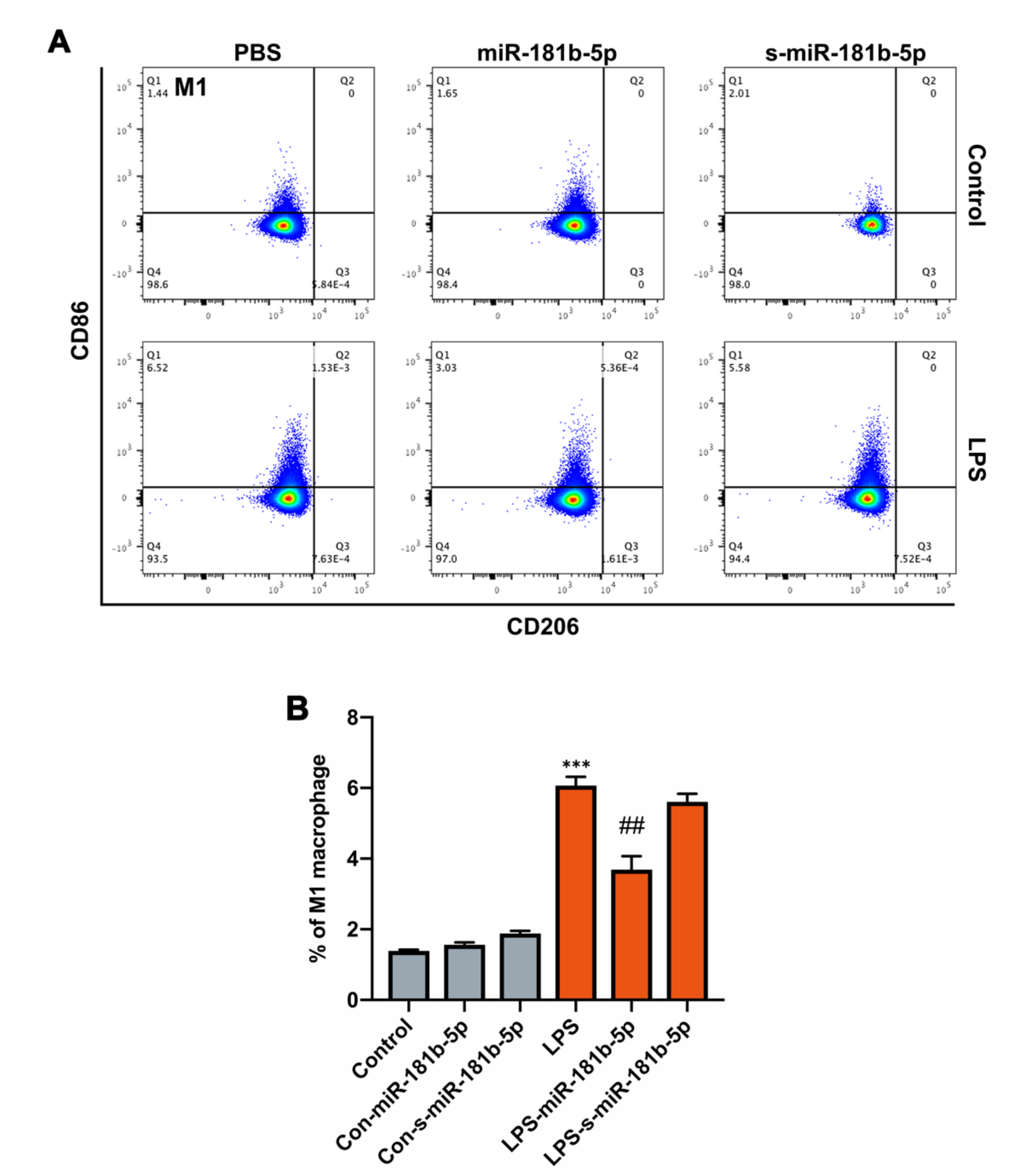

Figure S5 Effects of miR-181b-5p on M1/M2 macrophage number in RAW264.7 cells.

(A) Fluorescence activated Cell Sorting analysis of the RAW 264.7 cells stimulated by LPS (100ng/ml) with miR-181b-5p for 24 h. (B) Percentage of M1-type macrophage. Data were presented as the means ± SEM. *** $p < .001$, vs. Control; ## $p < 0.01$, vs. LPS.

Table S1 Primers sequences.

Gene name	Forward Primer	Reverse Primer
Rattus norvegicus (Norway rat)		
R-Muc1	5’-CTCCTTCTTCTCGTTGTCTT-3’	5’-GCTGCCTCCTTCTTATGTT-3’
R-Muc2	5’-GTGTTGTGGATGAGATGTTC-3’	5’-GGCGTTGACTTGGATGAT-3’
R-Muc3	5’-CCAAGGTGTGAGGAAGTG-3’	5’-GATGTTATCTAAGGTGCTGTC-3’
R-Tjp1	5’-TTCCTAAGACCTGTAACCATC-3’	5’-CATAGCCTCATCCTCATTGT-3’
R-Claudin1	5’-AAGATGTGGATGGCTGTC-3’	5’-TAGAAGGTGTTGGCTTGG-3’
R-Occludin	5’-TTGAGAGTCCACCTCCTTA-3’	5’-CACGATGACGAGCATAGA-3’
R-IL-6	5’-CAGAGTCATTCAGAGCAATAC-3’	5’-GATGGTCTTGGTCCTTAGC-3’
R-TNF-α	5’-AAGCATGATCCGAGATGTG-3’	5’-CCGAAGTTCAGTAGACAGAA-3’
R-NF-κB	5’-ATCCAACACAGGCATCAC-3’	5’-CCAGCAGCATCTTCACAT-3’
Mus musculus (house mouse)		
m-18s	5’-CGAACGTCTGCCCTATCAACTT-3’	5’-CCGGAATCGAACCCTGATT-3’
m-ZO-1	5’-AAGAATATGGTCTTCGATTGGC-3’	5’-ATTTTCTGTCACAGTACCATTTATCTTC-3’
m-CLDN-1	5’-GTTTGCAGAGACCCCATCAC-3’	5’-AGAAGCCAGGATGAAACCCA-3’
m-CLDN-2	5’-GTCATCGCCCATCAGAAGAT-3’	5’-ACTGTTGGACAGGGAACCAG-3’
m-CLDN-3	5’-GCTCTCAGAGTC CGTTGCC-3’	5’-CTGCCCTTTCAGGTTAGCAG-3’
m-MUC1	5’-AGTTACGGTCAGGCTGCTCCGTGGT-3’	5’-ACCCTCCCGGAAAACCACAGTC-3’
m-MUC2	5’-TGCTGCTGACGAGTGGTTGGTG-3’	5’-CGGACGCTTGGTGGTGAGGC-3’
m-Muc3	5’-TGGTCAACTGCGAGAATGGA-3’	5’-TACGCTCTCCACCAGTTCCT-3’
m-MUC4	5’-GGCCCGCTTGGACATTTGGTGA-3’	5’-AGTCTCCCAGCCCGTTGAAGGT-3’
m-lL-6	5’-CCACTTCACAAGTCGGAGGCTTA-3’	5’-GCAAGTGCATCATCGTTGTTCATAC-3’
m-TNF-α	5’-CCCACACCGTCAGCCGATTT-3’	5’-GTCTAAGTACTTGGGCAGATTGACC-3’
m-lL-1β	5’-GGGCCTCAAAGGAAAGAATC-3’	5’-TACCAGTTGGGGAACTCTGC-3’
m-CD206	5’-CAAGGAAGGTTGGCATTTGT-3’	5’-CCTTTCAGTCCTTTGCAAGC-3’
m-CD3	5’-GACTATGAGCCCATCCGCAA-3’	5’-AACAAGGAGTAGCAGGGTGC-3’
m-CD4	5’-TGTCACTCAAGGGAAGACGC-3’	5’-CGAAGGCGAACCTCCTCTAA-3’
m-CD8	5’-ACGAAGCTGACTGTGGTTGAT-3’	5’-AAAAGTAGACGGCCACTCCG-3’
m-IL-17α	5’-CCAAACACTGAGGCCAAGGA-3’	5’-TTCATTGCGGTGGAGAGTCC-3’
m-IL-22	5’-TGCAAGCTTGAGGTGTCCAA-3’	5’-AGCCGGACATCTGTGTTGTT-3’
m-NF-κB	5’-CCTGCAGGGTCACTCGATTT-3’	5’-TCAGAACCAAGAAGGACGCC-3’
m-F4/80	5’-GCTGTGAGATTGTGGAAGCA-3’	5’-CTGTACCCACATGGCTGATG-3’
m-JAM-A	5’-CACCTTCTCATCCAGTGGCATC-3’	5’-CTCCACAGCATCCATGTGTGC-3’
m-Gata3	5’-TCACACACTCCCTGCCTTCT-3’	5’-CACCCCATTACCACCTATCC-3’
m-Foxp3	5’-ATTGAGGGTGGGTGTCAGGA-3’	5’-CAGAGGCAGGCTGGATAACG-3’
m-Ccl5	5’-TGCCCTCACCATCATCCTCACT-3’	5’-GGCGGTTCCTTCGAGTGACA-3’
m-T-bet	5’-CCAAGACCACATCCACAAAC-3’	5’-CAACCAGCACCAGACAGAGA-3’
m-Defa3	5’-TCCTCCTCTCTGCCCTCGT-3’	5’-GACCCTTTCTGCAGGTCCC-3’
m-Defa20	5’-GACCTGCTCAGGACGACTTT-3’	5’-GCCTCAGAGCTGATGGTTGT-3’
m-TLR4	5’-GCAGAAAATGCCAGGATGATG-3’	5’-AACTACCTCTATGCAGGGATTCAAG-3’
Escherichia coli		
E-fimD	5’-CGGTATGAATCTGCTGGCGGATG-3’	5’-TGAGGGATCGTCAGGTTCAGTCG-3’
E-yieH	5’-GGTATCGACGCAGGTATGGAAGTG-3’	5’-GGTGGTGACTTTCGGGTGAACG-3’

E-atoB	5’-GGTGCCAGTGGTGCTCGTATTC-3’	5’-CCGTTCAATCACCATCGCAATTCC-3’
E-macB	5’-TCAGCCACACCTGCCGTCTC-3’	5’-AATCGCCGCTCACGCCATTG-3’
E-serB	5’-CGTGGCGATTGGCGATGGAG-3’	5’-CAGCGTGACGGATGGTGACTTC-3’
E-pdxK	5’-TCGGCACGGCAAAGACATTCAG-3’	5’-GCAGTCGCAGGTGGTTTACGG-3’
E-rulB	5’-ACTGGCGATAGACGGTGGATCTC-3’	5’-TGGAAAAGGCGGGCGATTTGG-3’
E-ung	5’-CGGCGTCACTATCTACCCAC-3’	5’-CTGGCCGAGAATCACCACTT-3’
E-pssA	5’-ATGAGGCTAAAAGGCAGCGT-3’	5’-TGGAACGCCATAAACCGGAA-3’
E-dcrB	5’-TATGACCGACCAGAGCGGTA-3’	5’-CCGCCAGATCTTCTTTCGGA-3’
E-ecnB	5’-GTGGCGTTGGTGAAGACATT-3’	5’-TATTGCTGCGCTTTCGTTGC-3’
E-glaH	5’-CTTCACCGAACAGACGACCA-3’	5’-CCGCTTGCTTCACATCATCG-3’
E-ynbD	5’-CATTACGCGAAGAGCAAGGC-3’	5’-TGCTCGTCTGTCAGCACAAT-3’
microRNA		
miR-200b-3p	5’-CGCAGTAATACTGCCTGGT-3’	5’-GGTCCAGTTTTTTTTTTTTTTTCATCA-3’
miR-181b-5p	5’-GAACATTCATTGCTGTCGGT-3’	5’-TCCAGTTTTTTTTTTTTTTTAACCCA-3’
miR-200b-5p	5’-GCATCTTACTGGGCAGCA-3’	5’-GTCCAGTTTTTTTTTTTTTTTCCAATG-3’
miR-26a-5p	5’-GCAGTTCAAGTAATCCAGGATAG-3’	5’-GTCCAGTTTTTTTTTTTTTTTAGCCT-3’
miR-215	5’-CGCAGCACTAGATTGTGAG-3’	5’-GTCCAGTTTTTTTTTTTTTTTCCAG-3’
miR-28-3p	5’-CGCAGATGACCTATGATTTGAC-3’	5’-GGTCCAGTTTTTTTTTTTTTTTGTCT-3’
U6	5’-CGCTTCGGCAGCACATAT-3’	5’-AGGTCCAGTTTTTTTTTTTTTTTA-3’
reverse primer	5’-CCAGTTTTTTTTTTTTTTTGGAAATCC-3’	

Table S3 miRNA and scramble miRNA sequences.

miRNA name	Sequence
miR-200b-3p	5’-UAAUACUGCCUGGUAAUGAUGA -3’
miR-200b-5p	5’- CAUCUUACUGGGCAGCAUUGGA -3’
miR-181b-5p	5’- AACAUUCAUUGCUGUCGGUGGGU -3’
miR-28b-3p	5’- CACUAGAUUGUGAGCUCCUGGA -3’
scramble miR-200b-3p	5’- GUUUAUUUAGCACUAGCAGAGA -3’
scramble miR-200b-5p	5’- UGCUUGUUAAUGAACGCGAGCC -3’
scramble miR-181b-5p	5’- GUUGUGCCAAAGCUAGUUGUCUG -3’
scramble miR-28b-3p	5’- CGCUAAGGAUUAUGUCCUGCAG -3’
ath-miR-416	5’- GGUUCGUACGUACACUGUUCA -3’
